# Nucleoporin NUP153 Phenylalanine-Glycine Motifs Engage a Common Binding Pocket within the HIV-1 Capsid Protein to Mediate Lentiviral Infectivity

**DOI:** 10.1371/journal.ppat.1003693

**Published:** 2013-10-10

**Authors:** Kenneth A. Matreyek, Sara S. Yücel, Xiang Li, Alan Engelman

**Affiliations:** Department of Cancer Immunology and AIDS, Dana-Farber Cancer Institute, and Department of Medicine, Harvard Medical School, Boston, Massachusetts, United States of America; University of Massachusetts Medical School, United States of America

## Abstract

Lentiviruses can infect non-dividing cells, and various cellular transport proteins provide crucial functions for lentiviral nuclear entry and integration. We previously showed that the viral capsid (CA) protein mediated the dependency on cellular nucleoporin (NUP) 153 during HIV-1 infection, and now demonstrate a direct interaction between the CA N-terminal domain and the phenylalanine-glycine (FG)-repeat enriched NUP153 C-terminal domain (NUP153_C_). NUP153_C_ fused to the effector domains of the rhesus Trim5α restriction factor (Trim-NUP153_C_) potently restricted HIV-1, providing an intracellular readout for the NUP153_C_-CA interaction during retroviral infection. Primate lentiviruses and equine infectious anemia virus (EIAV) bound NUP153_C_ under these conditions, results that correlated with direct binding between purified proteins in vitro. These binding phenotypes moreover correlated with the requirement for endogenous NUP153 protein during virus infection. Mutagenesis experiments concordantly identified NUP153_C_ and CA residues important for binding and lentiviral infectivity. Different FG motifs within NUP153_C_ mediated binding to HIV-1 versus EIAV capsids. HIV-1 CA binding mapped to residues that line the common alpha helix 3/4 hydrophobic pocket that also mediates binding to the small molecule PF-3450074 (PF74) inhibitor and cleavage and polyadenylation specific factor 6 (CPSF6) protein, with Asn57 (Asp58 in EIAV) playing a particularly important role. PF74 and CPSF6 accordingly each competed with NUP153_C_ for binding to the HIV-1 CA pocket, and significantly higher concentrations of PF74 were needed to inhibit HIV-1 infection in the face of Trim-NUP153_C_ expression or NUP153 knockdown. Correlation between CA mutant viral cell cycle and NUP153 dependencies moreover indicates that the NUP153_C_-CA interaction underlies the ability of HIV-1 to infect non-dividing cells. Our results highlight similar mechanisms of binding for disparate host factors to the same region of HIV-1 CA during viral ingress. We conclude that a subset of lentiviral CA proteins directly engage FG-motifs present on NUP153 to affect viral nuclear import.

## Author Summary

Lentiviruses such as HIV-1 possess mechanisms to bypass the nuclear envelope and reach the nuclear interior for viral DNA integration. Numerous nuclear transport proteins are important for HIV-1 infection, suggesting the viral nucleoprotein complex enters the nucleus by passing through nuclear pore complexes. HIV-1 was previously found to utilize cellular nucleoporin (NUP) 153 protein in a manner determined by the viral capsid protein. Here, we show HIV-1 capsid directly binds NUP153 in a phenylalanine-glycine motif-dependent manner; such motifs form the general selectivity barrier that restricts transport through the nuclear pore. We find that NUP153 binds a hydrophobic pocket found on capsid proteins from both primate and equine lentiviruses, suggesting an evolutionary predilection for this interaction. The pocket on HIV-1 capsid also binds phenylalanine moieties present in a small molecule inhibitor of HIV-1 infection, as well as a separate host factor implicated in the nuclear import pathway. We found that these molecules compete for NUP153 binding, providing insight into their mechanisms of action during HIV-1 infection. These results demonstrate a previously unknown interaction important for HIV-1 nuclear trafficking, and posit direct binding of viral capsids with phenylalanine-glycine motifs as a novel example of viral hijacking of a fundamental cellular process.

## Introduction

Retroviruses integrate their reverse transcribed genomes into host cell chromosomes to provide a permanent vantage from which to amplify themselves for subsequent transmission. As the nuclear envelope physically separates the host chromosomes from the cytoplasm during interphase, retroviruses have evolved mechanisms to bypass this natural barrier to the nuclear compartment. The γ-retrovirus Moloney murine leukemia virus (MLV) is believed to await the dissolution of the nuclear envelope during mitosis, a mechanism that limits infection by this virus to actively dividing target cells [1]–[3]. Lentiviruses such as HIV-1 infect post-mitotic cell subtypes during the establishment of host systemic infection, and correspondingly harbor mechanisms to infect cells during interphase, likely circumventing the nuclear envelope by passing through the channel present in the nuclear pore complex (NPC) [4], [5].

The vertebrate NPC is a large ∼120 MDa macrostructure, composed of ∼30 different proteins called nucleoporins (NUPs) that stack in rings of eight-fold symmetry to form the tubular pore as well as the attached cytoplasmic filaments and nuclear basket substructures [6], [7]. Approximately one-third of the NUPs harbor domains rich in phenylalanine-glycine (FG) motifs, commonly observed as FxF, FxFG, or GLFG patterns [8]. These FG-rich domains line the central channel of the NPC, as well as the cytoplasmic and nuclear openings [9], and dictate the selective passage of macromolecules through the pore; small molecules are able to passively diffuse, while molecules greater than ∼9 nm in diameter need to be ferried by specialized carrier proteins capable of interacting with the FG-based permeability barrier [10].

The HIV-1 nucleoprotein substrate for proviral integration, called the pre-integration complex (PIC), is estimated at ∼56 nm in diameter [11], and thus requires active translocation into the nucleus. While initial studies suggested that HIV-1 integrase (IN), matrix, and Vpr proteins, as well as a triple-stranded DNA structure of the reverse transcribed genome called the DNA flap, were key viral elements required for PIC nuclear import, subsequent studies found none of these factors to be essential [12]. Contrastingly, the viral capsid (CA) protein was shown to be the major viral determinant for infecting non-dividing cells [13], [14]. Various host proteins have also been shown to participate in HIV-1 nuclear import, with perhaps the most promising candidates emerging from a series of genome-wide RNA interference (RNAi) screens; factors identified in more than one of these screens include transportin-3 (TNPO3 or TRN-SR2), NUP358 (RANBP2), and NUP153 [15]–[17]. We have been particularly interested in NUP153, which plays an important CA-dependent role in HIV-1 PIC nuclear import [18], [19].

NUP153 is a FG nucleoporin that predominantly locates to the nuclear side of the NPC and exchanges dynamically with a nucleoplasmic population [20]. While NUP153 is anchored to the nuclear rim of the NPC through its N-terminal domain [21], its C-terminal FG enriched domain (referred to as NUP153_C_ herein) is natively unfolded and highly flexible [22]. The ∼200 nm long NUP153_C_ potentially reaches through to the cytoplasmic side of the NPC channel [23], shifting in spatial distribution in a transport-dependent manner [24], [25]. Human NUP153_C_ contains 29 FG motifs (FxF, FG, and FxFG patterns), which provide a vital role in NUP153-mediated nucleocytoplasmic transport [26]–[28].

While numerous studies have demonstrated the functional significance of CA for HIV-1 nuclear import and integration, the mechanistic details for these connections are incompletely understood. Retroviral CA proteins are composed of two α-helical domains, the N-terminal domain (NTD) and C-terminal domain (CTD), separated by a short flexible linker. CA multimerizes into hexameric arrays during particle maturation, while twelve interspersed pentamers dictate the overall shape of the condensed viral core [29]–[32]. While relatively intact cores enter the cell upon viral-cell membrane fusion, little if any CA remains associated with the PIC within the nucleus [33]–[37]. The precise location and mechanism of CA core disassembly remains controversial: while initial steps of core uncoating are tied to reverse transcription [38], subsequent events may involve binding to host proteins. This may involve cyclophilin A (CypA) and the NUP358 cyclophilin homologous domain (CHD), both of which bind the cyclophilin binding loop protruding from the top of the CA NTD [39], [40], or cleavage and polyadenylation specific factor 6 (CPSF6), which binds a hydrophobic pocket [41] located between α-helices 3 and 4 within the NTD. The small molecule PF-3450074 (PF74), which inhibits HIV-1 infection by destabilizing incoming CA cores, also engages this same pocket [42]. CA-containing protein complexes have been observed alongside the nuclear envelope [43], suggesting that the ultimate steps of core uncoating may occur at the nuclear periphery and/or during PIC nuclear transport.

Here, we find that the CA proteins from numerous lentiviruses, including HIV-1 and equine infectious anemia virus (EIAV), directly bind NUP153_C_, with subsequent mapping demonstrating the importance of individual FG motifs themselves. A panel of HIV-1 CA mutants highlights the importance of side-chains lining the CA NTD helix 3/4 hydrophobic pocket, and competition with both CPSF6 and PF74 support this as the site of NUP153_C_ binding. Correlation between NUP153 binding and dependence on endogenous NUP153 expression additionally support the relevance of this interaction during infection. HIV-1 CA mutant viruses N57A and N57D were defective for NUP153_C_ binding and acutely sensitive to the arrest of the cell-division cycle, with a significant correlation between cell cycle and NUP153 dependencies observed among an expanded set of CA mutant viruses. Our data support a model whereby partially uncoated cores directly engage NUP153 FG motifs within the NPC to affect HIV-1 PIC nuclear import.

## Results

### NUP153_C_ binds the NTDs of a subset of retroviral CA proteins

**Figure 1:**
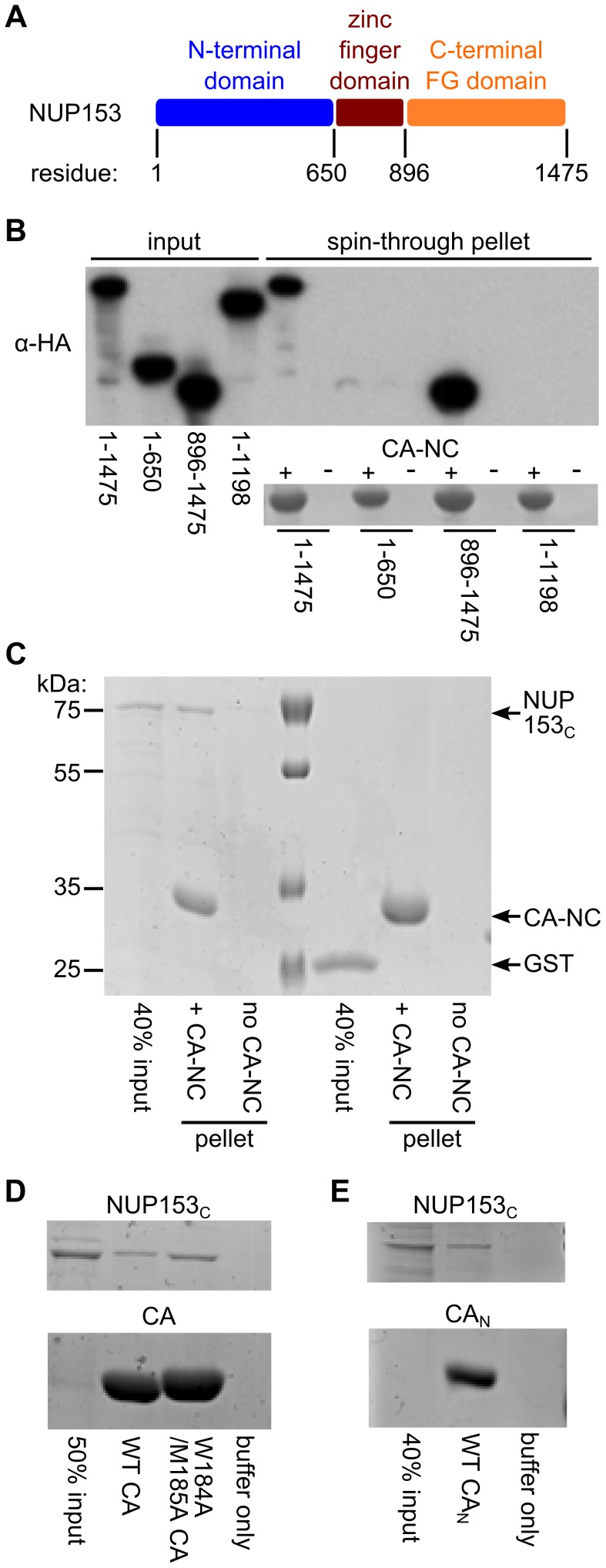
NUP153_C_ directly binds the HIV-1 CA N-terminal domain. (**A**) Schematic of NUP153 protein, with residue numbers of domain boundaries indicated. (**B**) Full length or truncated fragments of HA-tagged NUP153 extracted from 293T cells were tested for binding to HIV-1 CA-NC. Pelleted proteins were resolved by SDS-PAGE and visualized by western blotting with anti-HA antibody 3F10 (top), or by Coomassie stain (bottom). Input, 20% of binding reaction. CA-NC was included in the binding reactions as indicated. (**C**) Recombinant, tag-free NUP153_C_ and GST purified from *E. coli* were similarly tested for binding to CA-NC; proteins were detected with Coomassie stain. (**D**) Recombinant NUP153_C_ pulled down with full length his-tagged wild-type (WT) or W184A/M185A HIV-1 CA, and detected with Coomassie stain. (**E**) Recombinant NUP153_C_ pulled down with his-tagged CA_N_, and detected with Coomassie stain. Each experiment was repeated at least 3 times, with a single representative result shown.

As we previously found CA to be the dominant viral determinant of the requirement for NUP153 during HIV-1 infection [19], we tested whether a physical interaction between NUP153 and HIV-1 CA exists. Our initial assay utilized a recombinant viral fusion protein consisting of HIV-1 CA and nucleocapsid (NC) proteins, which when assembled in vitro in the presence of high salt and single stranded nucleic acid forms large tube-like structures that readily pellet through cushions of sucrose [29]. In this way, CA-interacting proteins can co-sediment with the tube structures [18], [44]. Full length or various fragments of HA-tagged NUP153 expression constructs were transfected into 293T cells, and the resulting proteins were tested for their ability to co-sediment with CA-NC assemblies. Full-length NUP153 (residues 1–1475) pelleted through the sucrose cushion in a CA-NC dependent manner (**Figure 1A and 1B**). The NUP153 N-terminal domain (residues 1–650) failed to bind CA-NC under conditions that supported efficient NUP153_C_ (residues 896–1475) binding. The C-terminal NUP153 deletion mutant comprised of residues 1–1198 failed to bind, confirming the importance of the NUP153 FG-repeat domain in binding, and mapping the interaction to residues 1199–1475 of the full length protein.

We addressed whether the NUP153–CA-NC interaction was the result of direct protein binding through the use of purified, recombinant NUP153 protein. We attempted to express full-length NUP153 fused to glutathione *S*-transferase (GST) in bacteria, but despite extensive effort, were unable to define conditions that yielded usable quantities of GST-NUP153 protein. Based on our preliminary binding data (**Figure 1B**), we instead expressed and affinity purified GST-NUP153_C_. NUP153_C_ was liberated from the GST tag by site-specific proteolysis, with the remaining CA binding studies utilizing tag-free NUP153_C_ protein. Approximately 40% of the input recombinant NUP153_C_ protein was recovered during co-sedimentation under conditions where binding of a negative control GST protein was undetected **(** **Figure 1C**). To test whether NUP153_C_ binds CA in the absence of NC and nucleic acid, his-tagged HIV-1 CA expressed and purified from *Escherichia coli* was utilized in Ni-nitrilotriacetic acid (NTA) pulldown assays. Approximately 30% of input NUP153_C_ was pulled down by his-tagged HIV-1 CA protein. Notably, this interaction is likely independent of CA oligomerization, as double mutant W184A/M185A CA, which is unable to dimerize and form higher-ordered assemblies [45], pulled-down comparable amounts of NUP153_C_ (**Figure 1D**). The isolated CA NTD (CA_N_) was expressed as a his-tagged protein and purified to next probe the binding region within HIV-1 CA; CA_N_ pulled down ∼30% of input NUP153_C_ protein (**Figure 1E**). Although these data do not quantitatively address potential CA oligomerization-based affects on NUP153_C_ binding, the relatively robust interaction with CA_N_ suggests that NUP153 may efficiently engage monomeric CA during HIV-1 infection.

**Figure 2:**
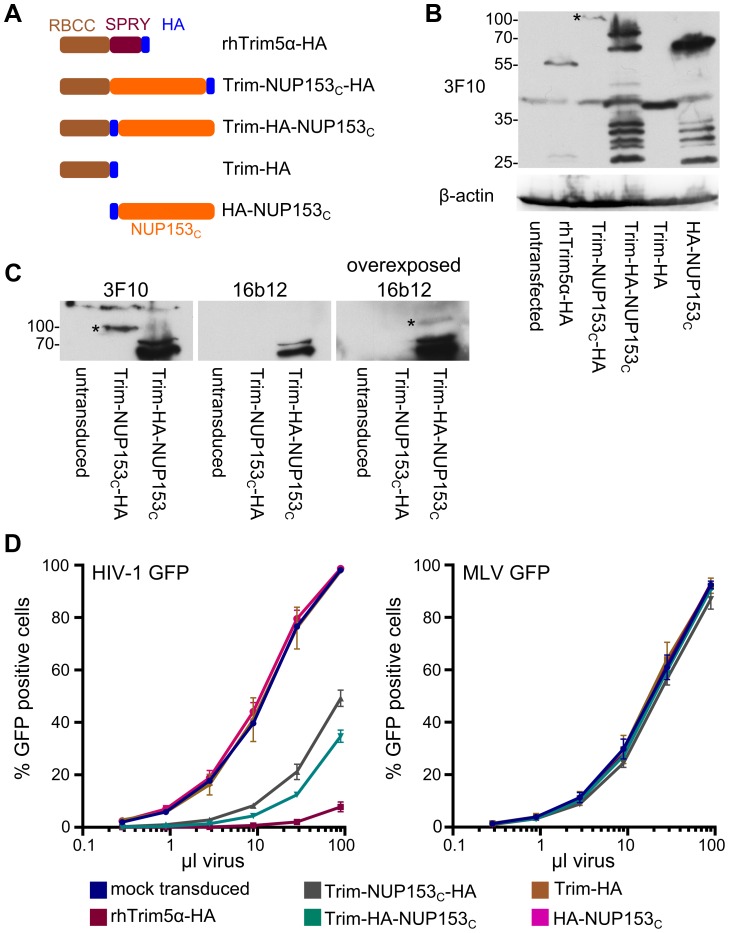
Restriction of HIV-1 infection by Trim5-NUP153_C_ fusion proteins. (**A**) Schematic of Trim-NUP153_C_ fusion and control constructs. Color code: Trim5 RBCC, brown; rhTrim5α SPRY, auburn; HA-tag, blue; NUP153_C_, orange. (**B**) Western blot of HOS cells stably transduced with HA-tagged Trim-NUP153_C_ fusion or control constructs, detected with antibody 3F10. (**C**) Western-blot detection of Trim-NUP153_C_ fusion proteins with anti-HA monoclonal antibodies 3F10 and 16b12. Antibody 3F10 detects full-length Trim-NUP153_C_-HA whereas antibody 16b12 more faithfully detects full-length Trim-HA-NUP153_C_. (**D**) Infectivity of various doses of HIV-1 (left) or MLV (right) GFP reporter viruses on HOS cells stably expressing various Trim-based constructs. The results are an average of two experiments, with error bars denoting standard error. Asterisks in panels B and C mark bands that correspond to the expected mobilities of full length Trim-NUP153_C_ constructs.

The preceding results established a direct interaction between NUP153 and HIV-1 CA proteins in vitro. We next examined whether an assay could be constructed to visualize the interaction in the context of HIV-1 infection. We scored for potential intracellular interaction by relying upon the potent capability of rhesus Trim5α (rhTrim5α) to inhibit HIV-1 infection. RhTrim5α is a cytoplasmically localized restriction factor, capable of blocking HIV-1 infection at an early post-entry step [46]. While the C-terminal B30.2 (SPRY) domain recognizes patterns present on the surface of retroviral CA cores [47], [48], the N-terminal RING, B-box 2, and coiled coil (RBCC) effector domains block infection by eliciting a combination of inhibitory activities, including premature disassembly of the viral core [44], proteasomal targeting [49], and triggering of innate immune signaling [50]. Both naturally occurring, as well as artificially engineered variants of Trim5 have been discovered wherein the SPRY domain is replaced by heterologous coding sequences, retaining viral restriction while changing the method by which the viral core is recognized [40], [51], [52]. In this vein we tested for intracellular recognition between NUP153_C_ and HIV-1 CA by replacing the SPRY domain of rhTrim5α with NUP153_C_, concomitantly introducing either an internal- or C-terminal HA epitope tag to enable detection of the fusion proteins by western blotting (**Figure 2A**). These constructs, as well as control constructs encoding only the epitope-tagged rhTrim5 RBCC or NUP153_C_, were stably introduced into human osteosarcoma (HOS) cells (**Figure 2B**). While a single species of C-terminally HA tagged Trim-NUP153_C_ of the expected molecular weight was detected by western blot, the internally tagged construct revealed the protein susceptible to degradation, with the full-length protein representing only a minority of the expressed products at steady state (**Figure 2B and 2C**). Regardless, Trim-NUP153_C_ expressing cells potently restricted HIV-1 infection, yielding consistent 5–10 fold reductions in viral titer (**Figure 2D**). The combination of both rhTrim5 RBCC and NUP153_C_ domains was necessary, as neither domain expressed alone inhibited HIV-1 infection. Knockdown of endogenous NUP153 acutely attenuates HIV-1 infection with little or no effect on MLV [19]. Importantly, the observed attenuation of HIV-1 infection by Trim-NUP153_C_ expression was specific, as infection by an MLV reporter virus was unaffected (**Figure 2D**). Similar to parental rhTrim5α, Trim-NUP153_C_ located to the cell cytoplasm (**Figure S1A and S1B**) and prevented HIV-1 from completing reverse transcription (**Figure S1C–F**), suggesting that it likely recognizes the HIV-1 CA core in the cytoplasm shortly after viral entry. We conclude that although NUP153_C_ in the context of the Trim5 protein likely engages HIV-1 CA earlier than endogenous NUP153 protein, the novel fusion nonetheless affords the analysis of the NUP153-CA interaction in the context of HIV-1 infection. Due to the marginally greater level of restriction imparted by the internally tagged construct, the Trim-HA-NUP153_C_ variant was used in subsequent experiments.

**Figure 3:**
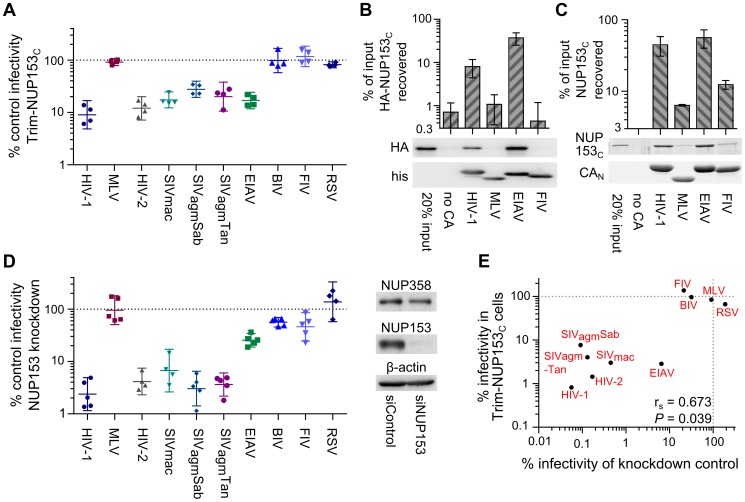
Diverse lentiviruses bind NUP153_C_. (**A**) Transduction efficiencies of retroviral GFP reporter viruses in Trim-NUP153_C_ expressing cells normalized to infection in mock transduced cells, which were set to 100%. Results are the geometric mean of 4 experiments, with error bars denoting 95% confidence intervals. (**B**) HA-NUP153_C_ expressed in 293T cells was pulled down by the indicated his-tagged retroviral CA_N_ proteins. Captured proteins resolved by SDS-PAGE were western blotted with antibody 3F10 alongside a standard curve of input protein. The results are an average of 5 experiments, with error bars denoting 95% confidence intervals. A representative western blot is shown. (**C**) SYPRO Ruby detection of retroviral CA_N_ pull-down of purified NUP153_C_. The results are an average of two experiments, with error bars denoting standard error; one representative gel is shown. (**D**) (left) Retroviral infectivities in HOS cells knocked down for NUP153 expression as compared to cells treated with a non-targeting short interfering (si) RNA control [19]. The results are the geometric mean of at least 4 experiments, with error bars denoting 95% confidence intervals. (right) Western blot detection of control or NUP153 knockdown HOS cells with antibody mab414, which also detects NUP358. (**E**) Scatter plot comparing relative retroviral infectivities under each condition.

Subjecting a panel of divergent retroviral reporter viruses to Trim-NUP153_C_ inhibition further validated the readout for intracellular CA core recognition. Primate lentiviruses SIVmac, SIVagmSab, SIVagmTan, and HIV-2 were similarly sensitive to Trim-NUP153_C_ inhibition (**Figure 3A**). Though EIAV was also sensitive, not all lentiviruses were: neither bovine immunodeficiency virus (BIV) nor feline immunodeficiency virus (FIV) was inhibited by Trim-NUP153_C_. The more distantly related α-retrovirus Rous sarcoma virus (RSV) was also unresponsive. To correlate the results of Trim-mediated restriction of virus infection to direct protein binding, a subset of the sensitive (EIAV) and nonresponsive (MLV and FIV) CA_N_ proteins was purified following their expression in bacteria. EIAV CA_N_ bound NUP153_C_ as efficiently as HIV-1 CA_N_, whereas binding to either MLV or FIV CA_N_ was significantly less efficient (*P*<0.01) **(** **Figure 3B and 3C**). Reliance on NUP153 during retroviral infection was compared with CA-NUP153_C_ binding (**Figure 3A**) by correlating percent infectivity in the face of NUP153 knockdown [19] (repeated here using HOS cells; **Figure 3D**). The resulting Spearman rank coefficient of 0.673 was statistically significant (*P* = 0.039) (**Figure 3E**).

### FG motifs within NUP153 dictate CA binding

**Figure 4:**
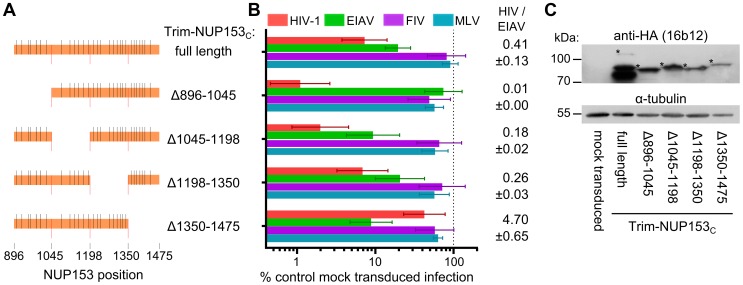
Different NUP153_C_ sub-regions mediate Trim-NUP153_C_ restriction of EIAV versus HIV-1 infection. (**A**) To-scale schematic of NUP153_C_ sequences encoded in various Trim-NUP153_C_ constructs. Red lines represent boundaries of quarter-sized NUP153_C_ sub-regions, while black lines denote the locations of FG motifs. (**B**) Infectivity of retroviral GFP reporter viruses on HOS cells stably expressing full-length or quarter-deleted Trim-NUP153_C_ constructs, normalized to infection in mock transduced cells. Data represent the geometric mean of 5 experiments, with error bars denoting 95% confidence intervals. HIV-1 to EIAV ratios of infectivity are shown, with associated standard error. (**C**) Western blot of HOS cells stably transduced with Trim-NUP153_C_ fusion constructs detected with antibody 16b12. Asterisks denote bands corresponding to the expected mobilities of full length or mutated Trim-NUP153_C_ constructs.

Mutations within NUP153_C_ were made to decipher the components of NUP153 critical for binding. As the HIV-1 restriction assay was higher throughput than the expression and purification of separate NUP153_C_ proteins, we first engineered mutations within the Trim-NUP153_C_ fusion construct. Since the starting fusion construct contained the entire ∼580 amino acid NUP153_C_, we generated cell lines stably expressing Trim fusion proteins with roughly quarter-size deletions of NUP153_C_, and determined the extent to which these constructs inhibited HIV-1 and EIAV infection, using MLV and FIV as negative controls (**Figure 4A and 4B**). Relative levels of HIV-1 and EIAV infection were compared to ease the interpretation of results to Trim-NUP153_C_ mediated restriction; parental Trim-NUP153_C_ yielded an HIV-1 to EIAV infectivity ratio of ∼0.41 (**Figure 4B**). Deletion of residues 896 to 1045 at the N-terminus of NUP153_C_ resulted in a construct that potently inhibited HIV-1 infection to a level ∼8 fold greater than the full-length construct, yet lost the ability to inhibit EIAV, yielding an HIV-1 to EIAV infectivity ratio of ∼0.01 (**Figure 4B**). Contrastingly, deletion of C-terminal residues 1350 to 1475 resulted in a protein still capable of inhibiting EIAV infection to a level comparable to the full-length construct, yet incapable of inhibiting HIV-1 infection beyond the level of the control viruses, resulting in an infectivity ratio of ∼4.70. These effects were specific to sequences deleted in the preceding constructs, as neither internal deletion noticeably perturbed the original Trim-NUP153_C_ restriction pattern; both constructs displayed the same slight advantage to inhibit HIV-1 infection over EIAV, with HIV-1 to EIAV infectivity ratios similar to the full-length construct. Western blotting confirmed that each deletion construct was expressed at roughly similar levels (**Figure 4C**). The mapping of the HIV-1 binding determinant on NUP153_C_ to residues 1350–1475 by Trim-mediated restriction notably coincides with our preliminary identification of the region C-terminal to residue 1198 using CA-NC tubes and HA-tagged NUP153 deletion constructs (**Figure 1B**).

**Figure 5:**
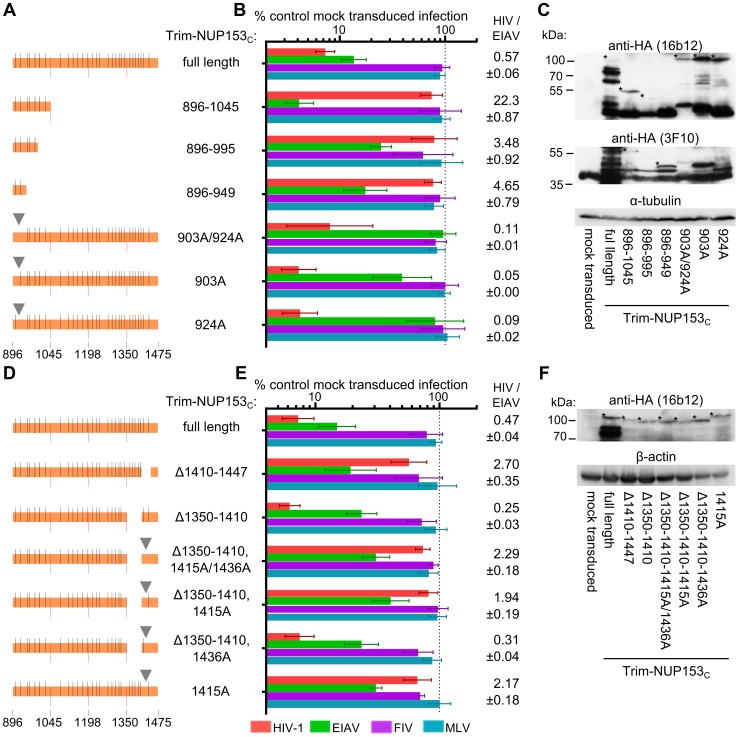
The importance of FG motifs for Trim-NUP153_C_ mediated inhibition of HIV-1 and EIAV infection. To-scale schematics (**A and D**), normalized infection data (**B and E**), and western blotting (**C and F**) as described for Figure 4. Infection data are the geometric mean of at least 4 experiments, with error bars denoting 95% confidence intervals. Inverted grey triangle (panels A and D) denotes area of missense mutation.

We next focused on the initial quarter of NUP153_C_ for its importance in mediating restriction of EIAV infection. Stable cell lines expressing only the first quarter of NUP153_C_ fused to the Trim RBCC, as well as smaller derivatives of the NUP153_C_ sequence, were generated (**Figure 5A**). Residues 896–949, which yielded the smallest construct capable of restricting EIAV infection (**Figure 5B**), harbored only two of the 29 FG motifs present within NUP153_C_. The importance of these FG motifs in mediating EIAV restriction was tested by substituting four consecutive alanine residues for each corresponding FKFG sequence. The combination octa-alanine 903A/924A Trim-NUP153_C_ mutant construct lost its ability to inhibit EIAV infection despite being expressed at a level equal to or greater than unmodified Trim-NUP153_C_ (**Figure 5B and 5C**). The 903A/924A mutant moreover retained potent HIV-1 restriction. Separate mutation of each motif revealed 924-FKFG-927 as the dominant FG sequence for mediating EIAV restriction.

Sequence components of NUP153_C_ that mediated restriction of HIV-1 infection were investigated next. Attempts to recover cells expressing the responsible C-terminal quarter of NUP153_C_ (residues 1350–1475) fused to Trim RBCC were unsuccessful. We instead undertook the alternative strategy to internally delete segments of residues 1350–1475 from the full-length Trim-NUP153_C_ construct (**Figure 5D**). Deletion of residues 1410–1447 selectively diminished inhibition of HIV-1 without affecting EIAV, yielding an increased HIV-to-EIAV infectivity ratio of 2.7, while deletion of residues 1350–1410 did not drastically alter the ratio from that observed with the full length construct (**Figure 5E and 5F**). As residues 1410–1447 contained only one FxFG and one FxF motif, these were mutated to alanine residues, initially in the context of the Δ1350–1410 construct. Combinatorial alteration of both tetra- and tri-peptides reduced restriction of HIV-1 without significantly affecting EIAV restriction (HIV-1/EIAV infectivity ratio = 2.29). Separate mutation showed this effect was largely, if not entirely due to 1415-FTFG-1418, and the 1415A mutation largely prevented restriction of HIV-1 in the full-length construct as well (infectivity ratio = 2.17). Combined, these results highlight the importance of FG motifs for Trim-NUP153_C_ mediated restriction of HIV-1 and EIAV infection. Moreover, different FG motifs appear to selectively recognize HIV-1 versus EIAV CA proteins.

**Figure 6:**
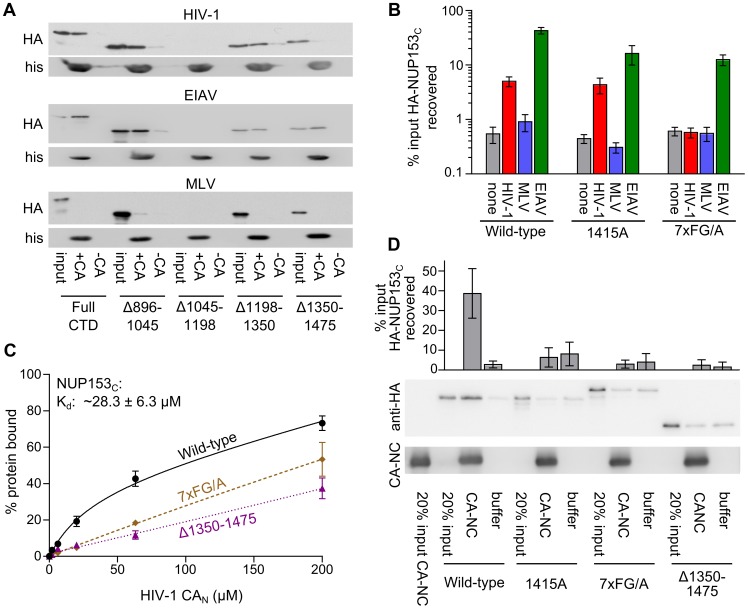
FG motifs determine NUP153_C_ binding to HIV-1 CA_N_. (**A**) Pull-down of full-length or quarter deleted HA-NUP153_C_ by HIV-1, EIAV, or MLV CA_N_ proteins, detected with antibody 3F10. (**B**) Pull-down of WT NUP153_C_, FG-motif tetra-alanine mutant 1415A, or combinatorial 7×FG/A mutant by beads alone (none, grey), HIV-1 (red), MLV (blue), or EIAV (green) CA_N_ proteins, as detected by western blot with antibody 3F10. Results are an average of at least 4 experiments, with error bars denoting standard error. (**C**) Purified NUP153_C_ (black circles, solid line), NUP153_C_Δ1350–1475 (purple triangles, fine dotted line), and NUP153_C_7×FG/A (brown diamonds, coarse dotted line) proteins were incubated with various concentrations of HIV-1 CA_N_ and a constant amount of Ni-NTA beads. Data points represent the mean and standard error of at least three experiments, fit with non-linear regression curves. The dissociation constant of NUP153_C_ binding was calculated by averaging concentrations of half-maximal binding for 5 individual experiments, with associated standard error. (**D**) Sedimentation of WT NUP153_C_, FG-motif tetra-alanine mutant 1415A, combinatorial 7×FG/A mutant, or NUP153_C_Δ1350–1475 after incubation with buffer alone or assembled CA-NC. Results are an average of 6 experiments, with error bars denoting 95% confidence intervals. Representative western blotting results are shown.

We subsequently tested for Trim-NUP153_C_ FG motif recognition of EIAV and HIV-1 CA proteins in vitro. HA-tagged NUP153_C_ or analogous quarter deleted fragments expressed in 293T cells were used as bait for pull-down by various his-tagged retroviral CA_N_ proteins (**Figure 6A**). The construct lacking residues 1045–1198 was expressed far less than the other constructs, and was not interpreted. As expected (**Figure 3B**), none of the constructs bound MLV CA_N_ to levels greater than those observed with beads alone. Consistent with the results from Trim-NUP153_C_ restriction, the protein lacking residues 1350–1475 was selectively bound less well by HIV-1 CA_N_. Contrastingly, EIAV CA_N_ bound all of the fragments tested, including the fragment that lacked residues 896–1045.

We further tested whether HIV-1 CA_N_ binding was traceable to specific FG motifs. HA-NUP153_C_ containing the 1415-FTFG-1418 tetra-alanine replacement bound HIV-1 CA_N_ essentially as well as the unmutated fragment (**Figure 6B**). Since we observed strongly diminished binding when the last quarter of HA-NUP153_C_ was deleted (**Figure 6A**), we next mutated all 7 of the FG motifs within this segment to alanines (HA-NUP153_C_7×FG/A). The combination of these mutations selectively abrogated binding of HA-NUP153_C_ to HIV-1 CA_N_; importantly, effective binding of the mutant protein to EIAV CA_N_ was retained (**Figure 6B**). Decreased Δ1350–1475 and 7×FG/A mutant binding to HIV-1 CA_N_ was also observed with purified NUP153_C_ proteins. HIV-1 CA_N_ bound purified NUP153_C_ (0.5 µM) in a dose-dependent manner, revealing a corresponding K_d_ of ∼28.3 µM at half-maximal saturation (**Figure 6C**). Although CA_N_ displayed some affinity for NUP153_C_Δ1350–1475 and NUP153_C_-7×FG/A, the shapes of these linear response curves were notably different from the unmutated protein, and half-maximal saturation was not reached under these assay conditions.

We hypothesized that differing states of CA multimerization might contribute to the partially overlapping specificities observed in the CA_N_ pull-down (**Figure 6A and 6B**) versus Trim-NUP153_C_ restriction (**Figure 5E**) assays. To test this, assembled CA-NC tubes were substituted for monomeric CA_N_ protein. Under these conditions, the 1415A mutant protein displayed significantly diminished binding, similar to the effects observed with the 7×FG/A and Δ1350–1475 mutant proteins (*P*<0.01) (**Figure 6D**). These findings seemingly agree with the results of the Trim-NUP153_C_ mediated restriction assays (**Figure 4B** **and** **5E**).

### Side-chains proximal to a common hydrophobic pocket in HIV-1 CA_N_ mediate NUP153_C_ binding

**Figure 7:**
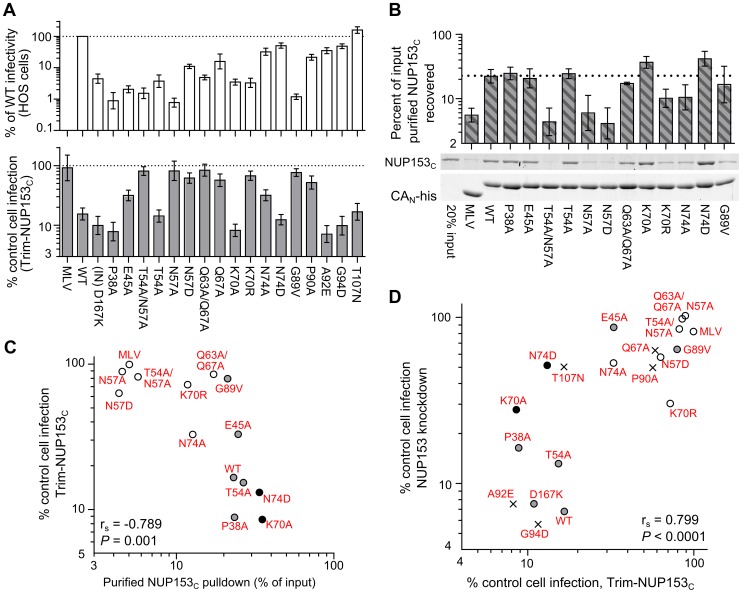
HIV-1 CA mutant-NUP153_C_ binding and sensitivity to Trim-NUP153_C_ restriction or NUP153 depletion. (**A**) (top) Equal reverse transcriptase (RT) cpm of WT and HIV-1 mutant viruses plated on HOS cells, with resulting infectivities normalized to WT virus. (bottom) Percent infectivity of viruses in Trim-NUP153_C_ expressing HOS cells, normalized to mock transduced control cells. Graphs show the mean of at least 5 experiments, with error bars denoting 95% confidence intervals. (**B**) Purified NUP153_C_ pull-down by WT or the indicated mutant his-tagged HIV-1 CA_N_ protein, with recovered proteins resolved by SDS-PAGE and detected by SYPRO Ruby stain. Results are an average of 5 experiments, with error bars denoting 95% confidence intervals. A representative staining result is shown. The dotted line highlights the level of NUP153_C_ binding to WT CA_N_ protein. (**C**) Scatter plot of NUP153_C_ recovery in pull-down assays (panel B) compared to percent infectivity in Trim-NUP153_C_ expressing cells (panel A, lower). Points are color-coded based on NUP153_C_ binding phenotype: grey, not significantly different from WT; white, significantly decreased from WT; black, significantly increased from WT. (**D**) Scatter plot of normalized infectivity of CA mutant viruses in Trim-NUP153_C_ expressing cells compared to the average infectivity of three experiments when endogenous NUP153 was knocked down. The comparison exhibited a significant Spearman rank correlation (*P*<0.0001). Points are color-coded as in panel C, except for CA mutants not tested for binding, which are denoted with “x” symbols.

We and others previously observed that various CA mutant viruses exhibit altered sensitivity to NUP153 knockdown [18], [19]. We next characterized an expanded set of CA mutant viruses for altered sensitivity to Trim-NUP153_C_ restriction. Mutants were selected based on prior descriptions of pre-integrative defects during HIV-1 infection. Alteration of CA residue(s) Pro38, Glu45, Thr54/Asn57, or Gln63/Gln67 can effect core stability [13], [38], [53], [54], whereas Thr54, Asn57, Lys70, Asn74, Gly89, Pro90, Ala92, Gly94, and Thr107 mutants can alter dependencies on various host proteins, including CPSF6, TNPO3, NUP358, CypA, or NUP153 [18], [19], [40], [41], [55]–[58]. As a number of these mutants exhibit drastically diminished overall levels of infectivity (**Figure 7A**, top), an unrelated IN mutant virus (D167K), which infects cells at ∼8% of the level of wild-type (WT) HIV-1 [59], was included to control for our ability to reproducibly measure restriction at reduced viral titers. While the IN mutant virus was as sensitive as the WT virus to Trim-NUP153_C_ restriction, a number of CA mutant viruses exhibited significantly reduced susceptibility (*P*<0.001) (**Figure 7A**, bottom). Included among these were CypA and NUP358 CHD binding mutants G89V and P90A [40], [55], as well as mutants E45A, T54A/N57A, N57A, N57D, Q63A/Q67A, Q67A, K70R, and N74A.

As these CA mutant viruses could resist Trim-NUP153_C_ restriction for any number of reasons, we tested for direct binding defects by pulling down NUP153_C_ with correspondingly purified CA_N_ mutant proteins. Residue Asn57 was critical for binding, as mutant proteins T54A/N57A, N57A, and N57D were strongly diminished in their abilities to pull down NUP153_C_ (**Figure 7B**). Although not critical for binding, both Lys70 and Asn74 appeared to participate: mutation of Lys70 to arginine diminished binding while mutation to alanine enhanced binding; contrastingly, mutation of Asn74 to alanine diminished binding, while mutation to aspartic acid enhanced binding to NUP153_C_. The Q63A/Q67A mutation marginally diminished binding by ∼1.3 fold. This binding hierarchy was also observed for HA-NUP153_C_ protein expressed in mammalian cells, with Asn57 again proving key for the interaction, and mutants K70A and N74D yielding hyper-binding activity (**Figure S2**). Overall, CA mutant viral sensitivities to Trim-NUP153_C_ restriction correlated well with CA_N_ mutant binding to NUP153_C_ protein in vitro (**Figure 7C**).

As we predict that mutant viruses that require NUP153 for infection also bind NUP153_C_, we compared the sensitivities of CA mutant viruses to NUP153 knockdown with their susceptibility to Trim-NUP153_C_ mediated restriction. We observed that CA mutant viruses that require endogenous NUP153 for infection were also sensitive to Trim-NUP153_C_ mediated restriction. A strong correlation supported this relationship across the entire panel of CA mutant viruses (**Figure 7D**). This included NUP153_C_ loss-of-binding mutants T54A/N57A, N57A and N57D, which retained approximately 85%, 102% and 58% of their infectivity, respectively, upon NUP153 knockdown.

### The NUP153_C_ binding site overlaps with those for PF74 and CPSF6

**Figure 8:**
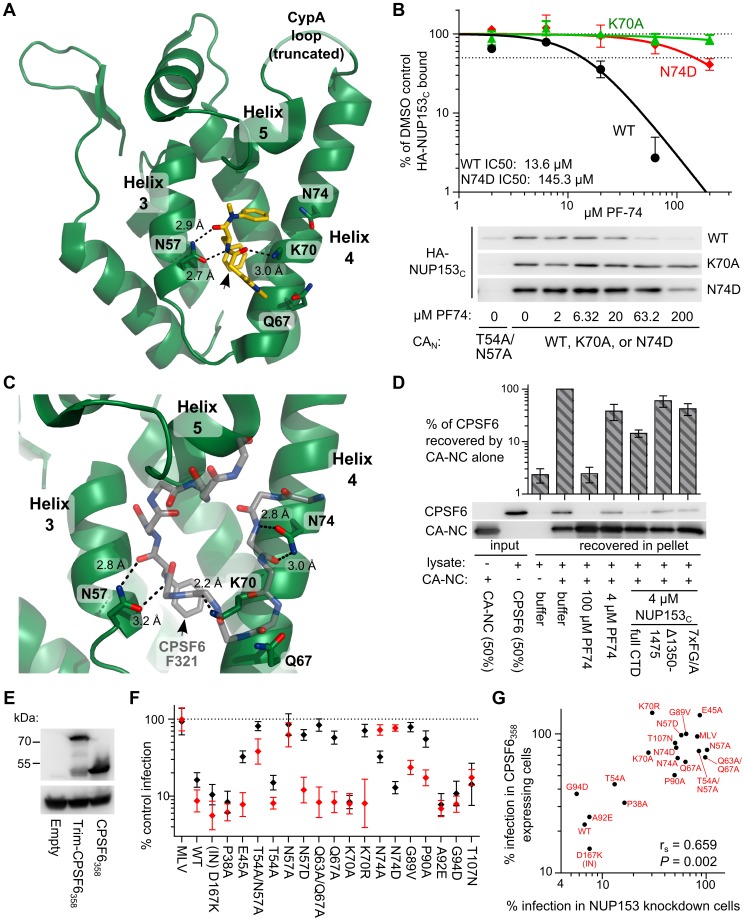
NUP153_C_ competes with molecules that bind the HIV-1 CA_N_ hydrophobic pocket. (**A**) X-ray crystal structure (pdb: 2×de) of compound PF74 (yellow) bound to HIV-1 CA_N_ (green). Critical CA_N_ side-chains (labeled) are shown as sticks, with oxygen and nitrogen atoms red and blue, respectively. Hydrogen bonds are shown as black dashes, with distances labeled. The phenylalanine moiety in PF74 is indicated by the black arrow. (**B**) PF74 competition of HA-NUP153_C_ binding to WT or mutant his-tagged HIV-1 CA_N_. Recovered HA-NUP153_C_ was detected with antibody 3F10 and quantitated alongside a standard curve of serially diluted HA-NUP153_C_-containing lysate. Baseline background signal observed with T54A/N57A CA_N_ was subtracted, and values were normalized to that of the DMSO control (2% DMSO final concentration in each sample). Results are an average of at least 2 experiments, with error bars denoting standard error. Representative western blotting results are shown. (**C**) X-ray crystal structure (pdb: 4b4n) of a peptide from CPSF6 (backbone carbon atoms shown as grey sticks) bound to CA_N_ (green) in the same orientation as in panel A. Side-chains and hydrogen bonds are represented as in panel A. The CPSF6 Phe321 side chain is indicated by the black arrow. (**D**) Binding of HA-tagged, full-length CPSF6 protein in 293T cell extract to HIV-1 CA-NC protein, and competition with purified NUP153_C_ or mutants thereof. Results of 5 experiments were normalized to the level of CPSF6 binding observed in the absence of competing factors, with error bars denoting standard error. (**E**) Western blot of HOS cells stably expressing Trim-CPSF6_358_ or CPSF6_358_, detected with antibody 3F10. (**F**) CA mutant virus sensitivities to Trim-NUP153_C_ (black) and Trim-CPSF6_358_ (red) restriction, as compared to cells transduced with an empty vector. Results are an average of at least 3 experiments, with error bars denoting 95% confidence intervals. (**G**) Scatter plot of CA mutant sensitivities to NUP153 knockdown compared with sensitivities to inhibition by CPSF6_358_.

Residues Asn57, Lys70, and Asn74, highlighted in our binding assays, surround a hydrophobic pocket within CA_N_ formed by α helices 3 and 4, and this pocket has been shown to be the binding site of the small molecule inhibitor PF74 [42] (**Figure 8A**). To probe potentially similar binding modes, we tested whether PF74 could compete for HA-NUP153_C_ binding to CA_N_ (**Figure 8B**). PF74 indeed competed for binding to CA_N_ in a dose-dependent manner, with an IC_50_ of ∼13.6 µM. While PF74 binds WT and N74D CA_N_ proteins similarly [41], the small molecule was less effective at competing for HA-NUP153_C_ binding to N74D CA_N_, yielding an IC_50_ of 145.3 µM, perhaps due to the increased binding observed between NUP153_C_ and N74D CA_N_ (**Figure 7B** **and S2**). PF74 does not bind K70A mutant CA_N_ [41], and accordingly did not compete for HA-NUP153_C_ binding to this mutant protein (**Figure 8B**).

This same pocket also engages the mRNA splicing cofactor CPSF6 [18], [41], [51], which was first implicated in HIV-1 biology by the ability for an exogenously expressed C-terminal truncation mutant CPSF6_358_ to restrict PIC nuclear import [18]. Though vastly differing molecules, co-crystal structures of PF74-CA_N_ and CPSF6 (residues 313–327)-CA_N_ complexes revealed that each exhibit nearly identical insertions of methyl benzyl residues (Phe321 in the case of CPSF6) within the helix 3/4 pocket, in both cases forming two hydrogen bonds with the carboxamide side-chain of CA residue Asn57 (**Figure 8A and 8C**). Based on these observations, we tested whether purified NUP153_C_ could compete with full-length CPSF6 protein for binding to CA. HA-tagged CPSF6 expressed in 239T cells was incubated with HIV-1 CA-NC tubes prior to centrifugation through a 20% sucrose cushion. CPSF6 pelleted only in the presence of CA-NC (**Figure 8D**). This interaction indeed required binding to the CA_N_ hydrophobic pocket, as excess PF74 counteracted it. We additionally observed that co-incubation with purified NUP153_C_ significantly diminished CPSF6 binding (*P*<0.0001) by ∼7 fold as compared to the level observed in the absence of competing factors. This competition was specific, as NUP153_C_ mutants Δ1350–1475 and 7×FG/A, both of which exhibit greatly diminished binding to CA-NC (**Figure 6**), were significantly less effective at competing for CPSF6 binding (*P*<0.05) (**Figure 8D**).

CA residues that mediate binding to NUP153_C_ and CPSF6 were further analyzed by assessing CA mutant sensitivities to restriction by the artificial restriction factor Trim-CPSF6_358_ (**Figure 8E**), a larger derivation of the Trim-CPSF6 fusion proteins previously tested [51]. Though conferring similar levels of restriction, far fewer of the CA mutant viruses were able to resist Trim-CPSF6_358_ inhibition (**Figure 8F**, red data points) compared to Trim-NUP153_C_ (black points). CypA binding mutants G89V and P90A were partially resistant to Trim-CPSF6_358_ restriction, whereas N57A, N74A, and N74D in large part conveyed full resistance. The N57A and N74D changes were notably previously shown to prevent binding of CA_N_ to the CPSF6 peptide [41]. Interestingly, changes at Asn57 and Asn74 conferred distinguishable resistance profiles to Trim-NUP153_C_ versus Trim-CPSF6_358_: both conservative N74D and non-conservative N74A changes rendered HIV-1 resistant to Trim-CPSF6_358_, while only N74A rendered the virus partially resistant to Trim-NUP153_C_ (**Figure 8F**). Contrastingly, both conservative and non-conservative Asn57 changes prevented Trim-NUP153_C_ recognition, while the conservative N57D mutant remained as sensitive to Trim-CPSF6_358_ restriction as the WT virus.

The breadth of CA mutants restricted by Trim-CPSF6_358_ in HOS cells appeared to contrast with prior results of CPSF6_358_-mediated restriction of HIV-1 in Hela cells, where many of the same CA mutations conferred resistance to inhibition [60]. We confirmed these phenotypes in HOS cells, where we observed that many additional CA mutant viruses resist CPSF6_358_-mediated restriction (**Figure S3**). Many of the CA mutant viruses selectively resistant to CPSF6_358_ over Trim-CPSF6_358_ restriction were also insensitive to endogenous NUP153 knockdown, resulting in a moderate correlation between CA mutant sensitivities to CPSF6_358_ restriction and NUP153 knockdown (**Figure 8G**).

**Figure 9:**
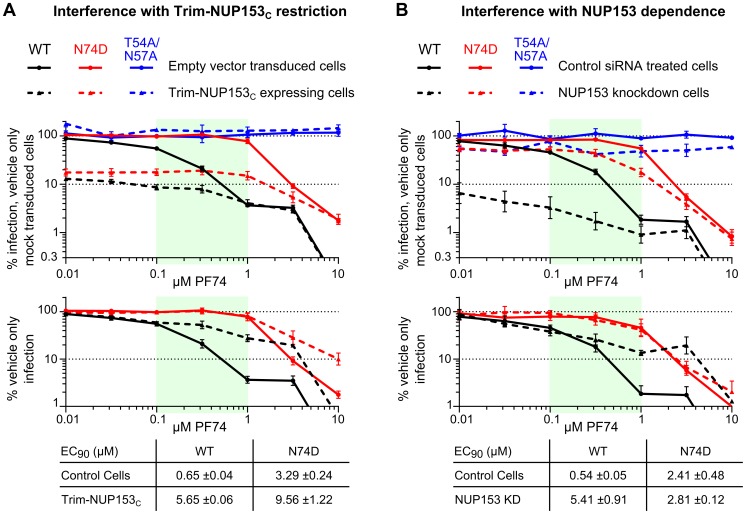
PF74 counteracts HIV-1 similarly in the face of Trim-NUP153_C_ restriction or NUP153 knockdown. Mock transduced and Trim-NUP153_C_ expressing (**A**) or non-targeting control and NUP153 knockdown (**B**) HOS cells were infected with equal RT-cpm of denoted viruses in the presence of various PF74 concentrations. Results are shown as infectivity normalized to vehicle only control cells (top), or vehicle only infection for each cell type (bottom) to calculate EC_90_ values. Dashed lines represent Trim-NUP153_C_ or NUP153 knockdown results in panels A and B, respectively. Results are an average of at least 3 experiments, with error bars denoting standard error. Calculated EC_90_ values are displayed with standard error.

PF74 destabilizes the structure of purified CA cores and can inhibit reverse transcription, which likely accounts for at least part of its antiviral activity [61]. We assessed whether PF74 could additionally antagonize NUP153_C_ engagement by CA in the context of HIV-1 infection, given the caveat that we could not unambiguously correlate data from protein binding assays (**Figure 8B**) with effects from PF74-induced capsid destabilization in cells. PF74 exhibited dose-dependent inhibition of WT HIV-1 and N74D CA mutant viral infection, but had no effect on CA mutant T54A/N57A, which lacks the critical Asn57 side-chain necessary for PF74 binding [41] (**Figure 9A** **, upper panel**; results replotted below to reveal EC_90_ values under conditions of Trim-NUP153_C_ restriction). WT virus was noticeably less sensitive to PF74 in Trim-NUP153_C_ expressing cells, with an EC_90_ of 5.65 µM as opposed to 0.65 µM in control cells (**Figure 9A**). The competing effect of PF74 on Trim-NUP153_C_ inhibition seemingly occurred between the concentrations of 0.1 and 1 µM (light green shading in Figure 9), as the inhibition curves within the two cell lines were nearly superimposable outside of these concentrations. N74D CA mutant virus also exhibited a shift in the PF74 EC_90_ concentration in Trim-NUP153_C_ cells, though this occurred at higher PF74 concentrations than with the WT virus. Interestingly, an almost identical effect was observed with WT virus when PF74 was titrated onto NUP153 knockdown cells; the EC_90_ shifted from 0.54 µM to 5.41 µM, with the same window of concentrations likely accounting for the discrepancy in inhibition curves (**Figure 9B**). While the exact mechanism of NUP153 antagonism – direct, or indirect through the alteration of the state of CA multimerization – is difficult to discriminate, the nearly superimposable interference profiles of PF74 in Trim-NUP153_C_ expressing and NUP153 knockdown cells support the relevance of the Trim-NUP153_C_ restriction assay as a surrogate readout for the engagement of endogenous NUP153 protein by the virus.

### An analogous pocket in EIAV CA mediates binding to NUP153_C_

**Figure 10:**
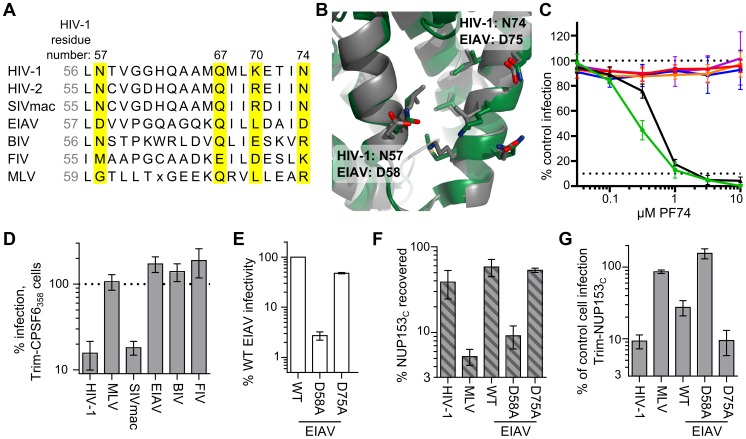
Mode of NUP153_C_ binding to EIAV CA. (**A**) Alignment of residues corresponding to HIV-1 CA Leu56 through Asn74 among various retroviruses. Residues that significantly affected HIV-1 CA_N_ binding with NUP153_C_ are highlighted in yellow. (**B**) Alignment of HIV-1 CA_N_ (green, pdb: 3mge) and EIAV CA_N_ (gray, pdb: 1eia), with side-chains surrounding the pocket shown as sticks. (**C**) Retroviral sensitivities to inhibition by PF74. Color codes: HIV-1, green; SIVmac, black; MLV, red; EIAV, orange; BIV, blue; FIV, purple. Results are an average of two experiments. (**D**) Retroviral sensitivities to inhibition by Trim-CPSF6_358_. Results are an average of 3 experiments. (**E**) Infectivity of RT-cpm matched EIAV GFP-reporter viruses carrying CA point mutations. Results are an average of 3 experiments. (**F**) Pull-down of purified NUP153_C_ by EIAV point mutant CA_N_ proteins. Results are an average of 2 experiments. (**G**) Sensitivity of EIAV CA point-mutant viruses to Trim-NUP153_C_. Results are an average of 4 experiments. Error bars in each panel denote standard error.

Retroviral CA_N_ proteins exhibit remarkable similarity in secondary and tertiary structure despite marked differences in primary sequence [62], [63]. With the exception of HIV-1 residue Gln67, the previously described polar residues flanking the helix 3/4 hydrophobic pocket (Asn57, Lys70, and Asn74 in HIV-1) exhibit variability across divergent retroviruses (**Figure 10A**, yellow boxes). While HIV-2 and SIVmac only differ at these positions with Arg69 in place of HIV-1 Lys70, EIAV exhibits greater difference: Leu71 corresponds to HIV-1 Lys70, and EIAV Asp58 and Asp75 correspond to HIV-1 Asn57 and Asn74, respectively (**Figure 10B**). These differences may account for the resistance of EIAV to inhibition by PF74 (**Figure 10C**) and CPSF6_358_ [51], which we confirmed using HOS cells expressing Trim-CPSF6_358_ (**Figure 10D**). As Asp58 exhibits similar physiochemical properties as its HIV-1 Asn57 counterpart, we mutated this as well as residue Asp75 to test their contributions to NUP153_C_ binding. Similar to HIV-1 mutant N57A, EIAV CA mutant D58A was poorly infectious (**Figure 10E**), and the corresponding CA_N_ protein was unable to pull down appreciable levels of NUP153_C_ protein (**Figure 10F**). Contrastingly, EIAV CA mutant D75A behaved similar to WT EIAV (**Figure 10E and 10F**). The Trim-NUP153_C_ sensitivities of these viruses corresponded with the binding profiles of their CA_N_ proteins: D58A was completely insensitive to Trim-NUP153_C_ mediated restriction, while D75A remained as sensitive as WT EIAV (**Figure 10G**).

### Comparison of NUP153 requirement and cell cycle dependence

**Figure 11:**
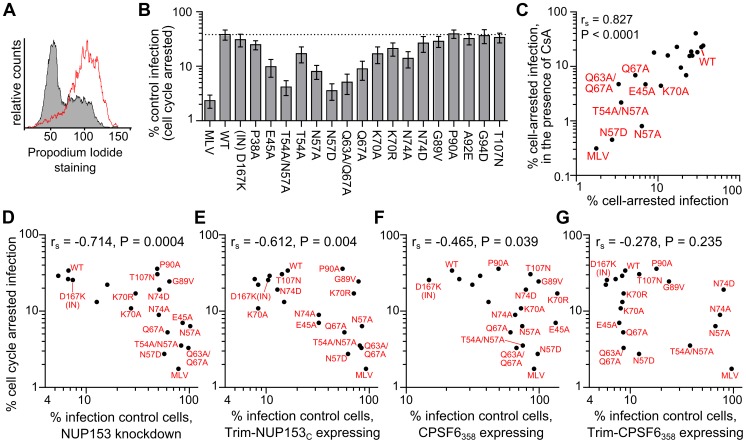
Association between NUP153 dependency and cell cycle independence. (**A**) Propidium iodide staining of HOS cells untreated (grey) or treated for 24 h with 5 µM Etoposide phosphate (red line). (**B**) Infectivity of CA mutant viruses in HOS cells arrested with 5 µM Etoposide phosphate, normalized to infectivity in control HOS cells. Error bars denote standard error of 4 experiments. (**C**) Scatter plot comparison of CA mutant sensitivities in cell cycle arrested HOS cells in the absence or presence of 5 µM cyclosporine (CsA). Mutant viruses most sensitive to cell cycle arrest are indicated. (**D to G**) Scatter plots comparing sensitivities of mutant viruses to cell cycle arrest versus NUP153 knockdown (D), or restriction by Trim-NUP153_C_ (E), CPSF6_358_ (F), or Trim-CPSF6_358_ (G). Spearman rank correlation coefficients and measures of significance are indicated. Data points for CA mutant viruses P38A, T54A, A92E, and G94D clustered with the WT virus within these panels, so their labels were omitted to aid legibility.

Changes at Asn57 in HIV-1 CA have previously been associated with cell cycle dependence: T54A/N57A infection was attenuated in both chemically arrested cell lines and non-dividing primary macrophages [13], [64], and the N57A mutant virus was recently shown to lose infectivity upon chemical arrest of Hela cells [40]. We confirmed the importance of Asn57, as well as other previously observed cell cycle dependent phenotypes, with our panel of CA mutant viruses; alanine substitution of residue Glu45, Thr54, Asn57, or Gln67 rendered the virus significantly sensitive to growth arrest (**Figure 11A and 11B**). Notably, we found even the conservative N57D substitution rendered the virus as, if not more sensitive, than these mutants to growth arrest. A handful of CA mutant viruses have been described to be sensitive to cell cycle arrest in Hela cells in a CypA-dependent manner [64], [65]. We found N57A and N57D CA mutant viruses to remain highly cell cycle dependent when the interaction with CypA was blocked by the addition of cyclosporine during infection (**Figure 11C**).

Based on the coincident NUP153-insensitive and cell cycle dependent phenotypes of Asn57 mutant viruses, we tested the association between NUP153 requirement and cell cycle dependency in the context of our expanded panel of mutant viruses. We observed a moderately strong inverse correlation between requirement for NUP153 and cell cycle dependence during infection (**Figure 11D**). Notably, of the viruses tested in our panel, all of the ones that were cell cycle dependent were NUP153 independent. The correlation however was not absolute, as N74D, G89V, P90A, and T107N mutant viruses did not require NUP153 for infection yet remained cell cycle independent. There was a moderate correlation between cell cycle dependence and Trim-NUP153_C_ resistance (**Figure 11E**). We observed a moderate to low correlation between cell cycle dependence and CPSF6_358_ mediated restriction, and no correlation with Trim-CPSF6_358_ mediated restriction (**Figure 11F and 11G**). These results reveal that cell cycle dependence is associated with NUP153 independence, and that this relationship likely depends on CA-NUP153 binding.

## Discussion

### NUP153 FG motif binding within the CA helix 3/4 cavity

Green fluorescent protein (GFP)-tagged NUP153 expressed in animal cell lysate was recently shown to co-sediment with HIV-1 CA-NC tubes in vitro [66]. We confirmed this observation for HA-tagged protein, and extended it by using purified recombinant protein to demonstrate direct binding between the FG-enriched NUP153_C_ and the HIV-1 CA NTD. Mutation of CA residue Asn57, Lys70, or Asn74, which each flank the hydrophobic pocket between CA α-helices 3 and 4, perturb binding of NUP153_C_ protein to HIV-1 CA_N_. Furthermore, NUP153_C_ competes with PF74 and CPSF6 for binding, both of which engage the same pocket. Notably, co-crystal structures between HIV-1 CA_N_ and the latter two molecules exhibit an almost identically situated benzyl ring within the hydrophobic cavity, with the amide nitrogen and carbonyl oxygens of this phenylalanine moiety each forming a hydrogen bond with the side chain of Asn57 [41] (**Figure 8**). This observation, in conjunction with our finding that FG motifs within NUP153_C_ strongly contribute to binding with CA_N_, suggest that the phenylalanine moieties of specific FG motifs found in NUP153_C_ likely take on a similar conformation during binding. We accordingly speculate that hydrogen bonding with Asn57 underlies the FG motif interaction, as both N57A and N57D mutations abrogated binding. While originally described to support CPSF6 binding [41], the high degree of amino acid conservation within this region of CA amongst primate lentiviruses likely also reflects the requirement for binding to NUP153 during virus infection [19].

### Relevance of FG motif binding for NUP153 dependency during HIV-1 infection

Supporting the relevance of the NUP153-CA interaction, both a divergent set of retroviruses and a targeted set of CA missense mutants exhibited significant correlations between CA binding to NUP153_C_ – either tested in vitro or inferred through Trim-NUP153_C_ recognition – and requirement for endogenous NUP153 protein during infection (**Figures 3** **and** **7**). Notably, loss-of-binding CA mutant viruses T54A/N57A, N57A, and N57D infected cells independent of endogenous NUP153 expression. The relationship between NUP153 binding and host factor requirement was consistent with PF74 sensitivity as well; while potentially mediated through an indirect effect on uncoating, PF74 interfered with Trim-NUP153_C_ restriction at the same concentrations that it antagonized the inhibition of infection caused by NUP153 knockdown (**Figure 9**).

Woodward and colleagues reported that ectopically-expressed NUP153_C_ protein imparted an approximate twofold defect on HIV-1 infection [67], a result we did not reproduce despite efficient NUP153_C_ expression (**Figure 2**). By contrast, appending NUP153_C_ to the RBCC domains of rhTrim5α resulted in potent HIV-1 restriction, allowing us to infer the results of NUP153_C_ binding to the CA shell during virus infection. NUP153 has been shown to bind HIV-1 IN [67], and though we observed minimal binding (≤1% of input IN recovered by GST-NUP153_C_ pull-down; **Figure S4**), it was comparably weaker than our findings with HIV-1 CA (30–40% of input NUP153_C_ recovered), and was less correlative with lentiviral requirement for endogenous NUP153 (**Figure 3D**) as FIV IN bound more robustly than HIV-1 IN to NUP153_C_ in our hands (**Figure S4**). Thus, while NUP153 may bind more than one HIV-1 determinant, our results are consistent with a direct interaction between NUP153_C_ and viral CA_N_ underlying the requirement for NUP153 during HIV-1 infection.

### Potentially degenerate binding of NUP153 FG motifs

Different FG motifs within Trim-NUP153_C_ mediated restriction of EIAV versus HIV-1 infection (**Figure 5**). Contrastingly, correspondence to protein binding in vitro was less strict: NUP153_C_Δ896–1045 effectively bound EIAV CA_N_, though this deletion variant could not inhibit EIAV as a Trim-fusion. The 1415-FTFG-1418 tetra-alanine mutant, which lost the ability to inhibit HIV-1 as a Trim-fusion, was little if at all reduced for pull-down by HIV-1 CA_N_, though alteration of all seven FG motifs in the last quarter of NUP153_C_ yielded a protein greatly deficient for binding to HIV-1 CA_N_ (**Figure 6**). Because the tetra-alanine 1415-FTFG-1418 NUP153_C_ mutant protein was significantly defective for binding assembled CA-NC tubes, we infer that this specific FG motif is particularly important for NUP153_C_ binding to multimerized CA.

We believe our results reflect the nature of the NUP153_C_-CA interaction during HIV-1 infection. Unlike a bimolecular interaction between two well-folded domains, each with a single binding site, NUP153_C_ exhibits no appreciable secondary structure and is highly repetitive in its primary sequence, particularly for phenylalanine-based FG motifs. As FG sequences appear to dictate NUP153_C_ binding to CA_N_, each of the 29 motifs may possess some affinity for CA_N_. Residues adjacent to the phenylalanine, such as glycine, may allow proper flexibility to fit into the helix 3/4 pocket for Asn57 engagement. We envision that residues peripheral to the motif may also contribute intra- and inter-molecular interactions. This interpretation is consistent with the mode of CPSF6 binding: the CPSF6 FG dipeptide (residues Phe321 and Gly322) is critical for CPSF6_358_ mediated restriction [51], while upstream residues Val314 and Leu315 fulfill important secondary roles through engaging additional hydrophobic patches located between CA_N_ helices 4 and 5. CPSF6 backbone functional groups also interact to varying degrees with the side-chains of CA residues Asn74, Thr107, Lys70, and Gln67 [41] (**Figure 8C**).

Given this model, we hypothesize that differential accessibility of the CA_N_ helix 3/4 pocket might factor into the contrasting binding specificities observed between monomeric and oligomerized CA: while the pocket is likely exposed as a soluble NTD fragment in the pull-down assay, it may be less available within the context of a multimeric CA array. The CTD of the adjacent CA subunit covers the bottom edge of the cavity (**Figure S5A**), and the interacting NUP153_C_ peptide would need to reach into the crevice between CA subunits, past the cyclophilin-binding loop, and under helix 5 to reach the pocket (**Figure S5B**). These steric requirements likely limit the number of NUP153_C_ FG motifs capable of forming energetically favorable interactions with the oligomerized CA array present on the viral core.

### Association with core uncoating and sensitivity to cell cycle arrest

Accordingly, alterations in the rate or extent of CA core uncoating may alter engagement of NUP153 during infection. Though both Trim-NUP153_C_ and Trim-CPSF6_358_ presumably encounter CA cores shortly after entry (**Figure S1**) [51], Trim-CPSF6_358_ restricted the hyperstable CA mutant viruses E45A and Q63A/Q67A [13], [38], [53], [54], [68] as efficiently as WT cores, while Trim-NUP153_C_ was less effective at restricting either of these mutants (**Figure 8F**). Both mutant CA_N_ proteins in large part retained NUP153_C_ binding in vitro (**Figure 7B**), suggesting that some CA disassembly may be needed for interaction with NUP153_C_ within cells.

These hyperstable CA mutant viruses acutely depend on the cycling state of the cell. Comparison between cell cycle dependence and NUP153 reliance resulted in a strong negative correlation within the panel of CA mutant viruses (**Figure 11D**). This correlation was stronger than the relationship between CPSF6_358_ sensitivity and cell cycle dependence (**Figure 11F**), suggesting a more direct association with NUP153 engagement. Consistent with this, the CPSF6 binding mutant N74D was cell cycle independent, while N57A and N57D mutant viruses, both of which are also defective for NUP153 binding, were sensitive. While the direct cause of cell cycle dependence is not clear, we suspect that defective NUP153 binding is a key contributor, and that hyper-stable CA cores may phenotypically mimic this effect.

### Competitors of NUP153-CA binding

The HIV-1 CA side-chains involved in NUP153_C_ binding overlap those identified to interact with CPSF6. Accordingly, we found recombinant NUP153_C_ able to compete with CPSF6 for binding to HIV-1 CA_N_ in vitro (**Figure 8D**). The overlapping binding sites suggest these proteins may take interdependent or even antagonistic roles during infection. While the role of endogenous CPSF6 protein in HIV-1 infection is unknown, the cytoplasmic CPSF6_358_ truncation variant potently restricts HIV-1 [18], [41], [51], [60], [69]. Like Trim-NUP153_C_, CPSF6_358_ may interact with the viral core shortly after entry; both a Trim-fusion protein containing the CPSF6 binding domain [51], and the cytoplasmically expressed CPSF6_375_ isoform [70], prevent the completion of reverse transcription. Interestingly, CPSF6_358_ does not inhibit reverse transcription, but instead blocks HIV-1 nuclear import. Additionally, CPSF6_358_ appears to inhibit only a subset of CA mutant cores that it is able to bind [60] (**Figure 8F** **and S3**). This may reflect an incomplete understanding of the mechanism of CPSF6_358_ restriction, which could involve antagonism of the CA-NUP153 interaction (**Figure 8G**). While CPSF6_358_-mediated stabilization of the CA core [60], [69] may contribute to the nuclear import defect, it seems possible that direct competition for NUP153 binding may also be at play.

Small molecules that bind the helix 3/4 pocket in CA may also preclude NUP153 binding during HIV-1 infection. At least part of the PF74 antiviral mechanism occurs before nuclear entry, as it can inhibit HIV-1 reverse transcription [61]. Yet, its altered dose-response curve in NUP153 depleted cells suggests that it antagonizes CA engagement of NUP153 as well (**Figure 9**). Notably, recently identified pyrrolopyrazolone small molecules BI-1 and BI-2 bind the same pocket, yet inhibit HIV-1 nuclear import [71]. As both PF74 and the pyrrolopyrazolone compounds bind CA_N_ with similar affinity [41], [71], we speculate that the contrasting phenotypes observed with these small molecules is due to their similar abilities to directly compete with host factors that bind the helix 3/4 pocket juxtaposed with their differential affects on CA core stability: PF74 destabilizes incoming capsids [61], whereas BI-1 and BI-2 can stabilize capsid structures in vitro [71]. TNPO3 depletion is proposed to mis-localize endogenous CPSF6 into the cytoplasm, recreating the phenotypes conferred by CPSF6_358_ expression [60]. Resembling our observations with NUP153 knockdown cells, infection of TNPO3 depleted cells exhibited a similar profile of reduced sensitivity to PF74 [72].

### Shared dependency profiles between host-factor binding mutants

A number of HIV-1 CA mutant viruses beyond the above noted hyperstable mutants exhibited resistance to NUP153 knockdown while maintaining near WT levels of protein binding. Many of these are defective for binding to other HIV-1 CA interacting host factors [40], [41]. Interestingly, the N74D mutant is defective for CPSF6_358_ binding [18], [41] but is competent to bind both NUP153 (**Figure 7B**) and NUP358 [40], yet requires neither of these nucleoporins for infection. Similarly, CA mutant virus N57A is NUP153 and CPSF6 binding-defective, detectably binds the NUP358 CHD, yet does not require NUP358 expression for infection [40]. The CypA and NUP358 CHD binding mutants G89V and P90A are also comparably less sensitive to NUP153 depletion and CPSF6_358_ restriction [19], [40], [60]. Though the mechanistic reasons for these relationships are unclear, loss of binding to one of these factors appears to render HIV-1 independent of the others.

### Model of NUP153 FG engagement during lentiviral infection

**Figure 12:**
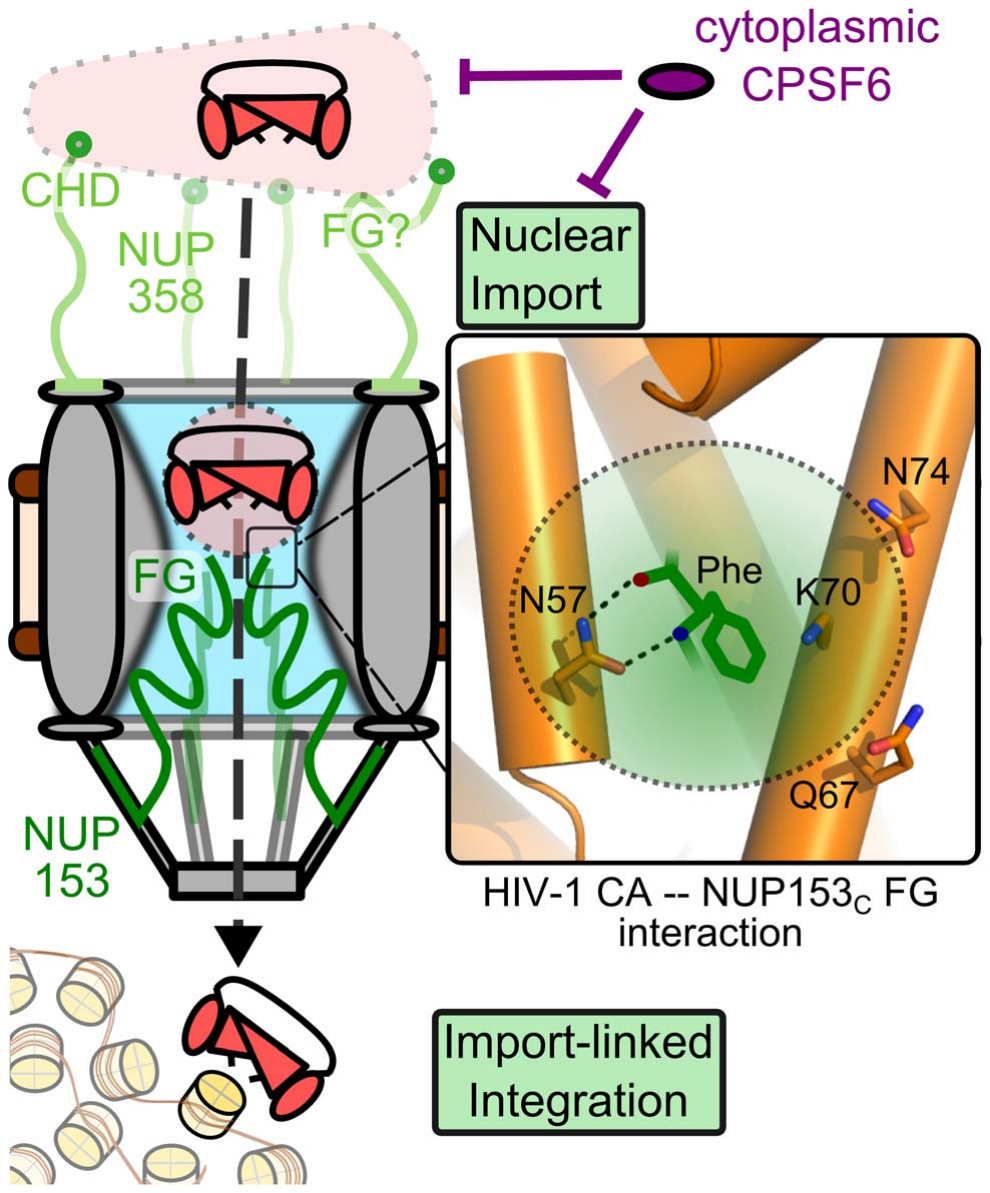
The NUP153-CA interaction during HIV-1 infection. Partially uncoated HIV-1 cores dock at the NPC through engaging NUP358 (light green). Once docked, NUP153 (dark green) FG motifs bind CA through phenylalanine insertion into the hydrophobic pocket of the NTD, forming hydrogen bonds with CA residue Asn57, as well as adjacent polar side-chains (enlarged to the right). CA engagement with NUP153 is required for HIV-1 nuclear import, either directly during PIC translocation, or for completion of a prerequisite uncoating step. Perturbation of NUP153 engagement may affect multiple steps, such as intranuclear trafficking and integration site selection. Cytoplasmic CPSF6 may inhibit HIV-1 nuclear import by antagonizing NUP153 binding to CA.

Current data and the known biology of this protein suggest NUP153 is likely important for trafficking the HIV-1 PIC through the nuclear pore and into the nucleus [16], [19], [73] (**Figure 12**). The viral nucleoprotein complex is likely to initially dock to the NPC by engaging NUP358 [73] through its CHD [40], though additional determinants of NUP358 engagement [74], such as FG motifs, may also participate. While intact HIV-1 cores are too large to enter the central channel, CA cores in various stages of disassembly may enter far enough for remaining CA to be accessed by the FG domains present in NUP153_C_. Bypassing NUP153 affects downstream steps of infection, including integration, likely by altering the chromosomal environment encountered by the PIC; NUP153 depletion or the N57A CA mutation shifts integration events away from gene-dense regions of chromatin [40], [66], [75], similar to the effects observed from NUP358 or TNPO3 knockdown, or the N74D CA mutation [40], [76].

CA binding with NUP153_C_ may serve two distinct roles during infection. Firstly, NUP153 may be responsible for physically translocating the PIC by engaging CA molecules that may associate with it. The relatively short half-life of NUP153 at the NPC may contribute to the release of the PIC into the nucleoplasm [20]. Secondly, as even a partially disassembled core could remain too large to efficiently pass through the NPC channel, CA interaction with NUP153 may be required to fully uncoat the viral core at the NPC and prime the PIC for nuclear import. Indeed, CA cores have been shown to dock to NPCs for several hours before PIC nuclear translocation [43]. CA oligomers may interact with a limited subset of NUP153_C_ FG motifs, while increased CA pocket accessibility from progressive core disassembly may expose monomeric CA to an expanded number of NUP153_C_ FG repeats. While CA mutant viruses such as N74D may uncoat differently and circumvent this mechanism without penalty in various transformed cell lines, they apparently incur steep costs to infectivity in other cell types, such as primary macrophages [40], [77].

### Convergence in NUP153 use amongst viral families

Divergent viruses have adapted to use NUP153 for their own devices. Our results suggest EIAV, which presents different amino acid residues flanking the CA_N_ hydrophobic pocket, may have either retained, or convergently evolved NUP153 binding. Hepatitis B virus (HBV) has also been reported to bind NUP153 during its nuclear transport; though the HBV core is sufficiently small to traverse the NPC channel, NUP153 binding is believed to be important for HBV core conformational change and genome release within the nuclear basket [78]. This interaction may also require binding to NUP153 FG motifs, as both of the broadly defined regions mapped for HBV capsid binding overlapped parts of NUP153_C_. The *S. pombe* homolog of NUP153, Nup124p, is important for Tf1 retrotransposition and binds the Tf1 Gag protein, though binding did not necessarily appear to map to Nup124p FG motifs [79], [80]. Perhaps akin to effects caused by differential HIV-1 uncoating, the requirement for Nup124p appears to be related to the state of Tf1 Gag multimerization [81].

It remains to be determined whether FG motifs found on additional nucleoporins may bind HIV-1 and aid its infection. While the effects of NUP98 depletion on HIV-1 infection are relatively modest [18], [66], [82], this protein can also co-sediment with HIV-1 CA-NC tubes in vitro [66]. Similarly, the GLFG-motif enriched domain of *S. cerevisiae* NUP100, predicted to be orthologous to vertebrate NUP98, binds Ty3 Gag protein [83]. Alternatively, while CA binding with the CHD is proposed to determine the requirement for NUP358 [40], it remains to be seen whether its own FG domains may bind CA and contribute to its function during infection. While numerous FG nucleoporins exist, it is likely that certain characteristics specific to NUP153, including its length, flexibility, and its relatively high dissociation rate from the NPC, along with its spatial location around the nuclear rim of the NPC, makes this protein particularly important for lentiviral passage through the nuclear pore.

## Materials and Methods

### Plasmid constructs

Infection assays utilized single-round viruses carrying either GFP or luciferase reporter genes. GFP-based constructs included HIV-1, EIAV, BIV, RSV, FIV, MLV, HIV-2 strain ROD, simian immunodeficiency viruses from *Macaca mulatta* clone 239 (SIVmac), *Chlorocebus sabaeus* (SIVagmSab), and *Chlorocebus tantalus* (SIVagmTan), all described previously [19], [84]. HIV-1 CA mutations were generated through site-directed mutagenesis of the HIV-1_NL4-3_-based pHP-dI-N/A packaging plasmid [85] (AIDS Research and Reference Reagent Program [ARRRP]), which were co-transfected with either pHI-vec2.GFP or pHI-Luc transfer vectors [19].

Human NUP153 (accession number NM_005124.3), or deletion mutants thereof, fused to N-terminal HA tags were expressed from the pIRES-dsRed Express HA-NUP153 expression vector [19]. Trim-fusion constructs, which were built within pLPCX-rhTrim5α-HA [46], were created by engineering a BamHI site at nucleotides corresponding to residues 301 and 302 of rhesus Trim5α, and ligating the digested vector with sequences encoding HA-NUP153_C_, NUP153_C_-HA, or CPSF6_358_-HA [18]. Truncated Trim-HA was engineered by modifying the Trim-HA-NUP153_C_ vector to encode two stop codons at the nucleotides corresponding to the first two residues of NUP153_C_. All deletion and missense mutations within animal-cell expressed NUP153_C_ were engineered by site-directed-mutagenesis of plasmids pLPCX-Trim-HA-NUP153_C_ or pLPCX-HA-NUP153_C_.

HIV-1_NL4-3_ CA carrying C-terminal his and FLAG tags was expressed from the pET11a-HIV1-CA-his-flag bacterial expression vector. The vector encoding tagged HIV-1 CA NTD (pET11a-HIV1-CA_N_-his-flag) was constructed by removing nucleotides corresponding to CA residues 147–231 from the full-length expression vector. Bacterial expression vectors for FIV CA were generated by amplifying DNA encoding full-length FIV CA (residues 1–223; pET11a-FIV-CA-his) or NTD only (residues 1–140; pET11a-FIV-CA_N_-his) from pFP93 [86] with a primer encoding a C-terminal his-tag, and ligating with digested pET11a DNA. pET22b-based bacterial expression vectors encoding C-terminally his-tagged N-tropic MLV (pET22b-NMLV-CA-his) and EIAV (pET22b-EIAV-CA-his) were obtained from the laboratory of Dr. Joseph Sodroski, and CA NTDs were engineered from full-length his-tagged constructs by removing nucleotides corresponding to residues 133–263 of N-MLV, and residues 149–231 of EIAV, by site-directed mutagenesis.

The construct pGEX2T-GST-NUP153_C_, which encoded GST fused to NUP153_C_, was created by deleting sequences that encoded residues 1–895 from the full-length human protein pGEX2T-hNUP153 bacterial expression vector [87]. Plasmid pGEX2T-his-GST-pp-NUP153_C_, which was utilized to obtain tag-free NUP153_C_ protein, was derived from pGEX2T-GST-NUP153_C_ by sequentially engineering a PreScission protease site between GST and NUP153_C_, and then appending a his-tag N-terminal to GST. A stop codon was introduced at the nucleotides corresponding to residue 1350 to generate the Δ1350–1475 truncation mutant. The 7×FG/A mutant NUP153_C_ was engineered for bacterial expression by swapping the WT sequence present in pGEX2T-his-GST-pp-NUP153_C_ with a fragment encoding NUP153 residues 1178–1475 amplified from pLPCX-HA-NUP153_C_-7×FG/A. All coding sequences were verified through DNA sequencing.

### Cells

293T and HOS cells were cultured in Dulbecco's modified Eagle's medium (DMEM) (Invitrogen) supplemented with 10% fetal bovine serum (FBS), 100 U/ml penicillin, and 0.1 mg/ml streptomycin. HOS cells stably transduced with MLV-derived LPCX transfer vectors were subsequently selected and maintained with 2 µg/ml puromycin. Approximately 25,000 HOS cells seeded per well of a 24-well plate were transfected the next day with a final concentration of 40 nM siNUP153#1 (GGACTTGTTAGATCTAGTT) or a mismatch control of siNUP153#1, referred to as siControl (GGTCTTATTGGAGCTAATT) (Dharmacon) [19], using RNAiMax (Invitrogen) according to the manufacturer's instructions. Dividing or cell cycle arrested cells were collected at the time of infection, fixed in 70% ethanol, and incubated for 30 min at room temperature in staining solution [0.1% Triton X-100, 0.2 mg/ml RNAse A (Invitrogen), and 20 µg/ml propidium iodide in phosphate-buffered saline (PBS)]. The cells were washed, and cellular DNA content was assessed with a FACSCanto flow cytometer (Becton, Dickenson and Company) equipped with FACSDIVA software.

### Virus production

Viral vector particles were produced by transfecting 293T cells in 10-cm plates with 10 µg total of various ratios of the aforementioned virus production plasmids using CaPO_4_. The cells were washed 16 h after transfection, and supernatants collected from 24 to 72 h thereafter were clarified at 300× g, filtered through 0.45 µm filters (Nalgene), and either allotted and frozen or concentrated by ultracentrifugation using an SW32Ti rotor at 50,000× g for 2 h at 4°C before freezing. Concentrations of HIV-1 and EIAV CA mutant viral stocks were determined alongside concomitantly produced WT viruses using an exogenous ^32^P-based assay for RT activity [88].

### Infectivity assays

HOS cells (10,000 or 2,500) seeded onto 48-well or 96-well plates, respectively, were infected with various reporter viruses. Percentages of GFP-positive cells were determined 48 h post-infection (hpi) using a FACSCanto flow cytometer equipped with FACSDIVA software. GFP reporter experiments comparing retroviral genera were performed with virus inoculates adjusted to yield ∼40% GFP-positive cells in control samples. HIV-1 or EIAV CA mutant viruses (2×10^5^ RTcpm) were used to infect 96-well and 48-well plates of cells, respectively. HIV to EIAV infectivity ratios were calculated after initially normalizing to the average of MLV and FIV negative control viruses to account for slight differences in overall infectivities between stable cell lines. Cyclosporine (5 µM, Sigma) was introduced to cells at the time of infection. Cell cycle arrest experiments were performed by plating 2,500 control or 5,000 experimental cells treated with 5 µM Etoposide-phosphate (Calbiochem) the day before infection. Quantitative PCR for the accumulation of viral late reverse transcripts and 2-long terminal repeat (LTR)-containing circles were performed as previously described [19]. The quantitation of early reverse transcripts was performed using primers AE989 and AE990 and Taqman probe AE995 [89].

### Western blot analysis

Cells stably expressing Trim-fusion proteins were lysed in Buffer A [25 mM Tris-HCl pH 7.5, 200 mM NaCl, 1 mM DTT, 1 mM EDTA, Complete protease inhibitor (Roche)] and sonicated for 30 s total with a misonix sonicator. Protein concentration of the bulk lysate was determined by Bradford assay (Bio-rad), and 75 µg of each sample were electrophoresed through Tris-glycine polyacrylamide gels, and transferred onto polyvinylidene fluoride membranes. Transiently expressed HA-tagged proteins were either extracted with buffer H [10 mM Tris-HCl pH 8.0, 10 mM KCl, 1.5 mM MgCl_2_] followed by repeated freeze-thaws, or Triton buffer [50 mM Triethanolamine, 250 mM NaCl, 0.5% Triton X-100], and pelleted in a microcentrifuge for 20 min at 21,000× g at 4°C. Stably expressing cells were also fractionated by initial lysis in Buffer F1 [20 mM Tris-HCl pH 7.5, 10 mM NaCl, 1.5 mM MgCl_2_, 0.25% Triton X-100, and Complete Protease Inhibitor], followed by centrifugation at 6,000× g. The supernatant was removed as Fraction 1, and the process was repeated, with the resulting supernatant combined with the previous fraction. The subsequent pellet was resuspended in Buffer F2 [Buffer F1 lacking Triton X-100, but with 0.5% sodium deoxycholate and 1% Tween-40], and pelleted at 21,000× g for 15 min. The supernatant was removed as Fraction 2, and pellet was resuspended in 1× Turbo DNase buffer and treated with 40 U/ml Turbo DNase (Ambion) for 10 min at 37° C. Two parts fraction 1, one part fraction 2, and one part of the remaining fraction (Fraction 3) were each mixed with sample loading buffer and separated on Tris-glycine polyacrylamide gels. Exogenously expressed HA-tagged proteins were detected using a 1∶4,000 dilution of HRP-conjugated 3F10 antibody (Roche) or 1∶4,000 dilution of mouse 16b12 antibody (Covance) and developed with ECL prime (GE Healthcare) or Femto (Thermo Scientific) detection reagents. NUP153, NUP62, and NUP358 were detected with a 1∶4,000 dilution of mouse monoclonal antibody mab414 (Abcam). HRP-conjugated mouse anti-β-actin antibody or mouse anti-α-tubulin antibody (Abcam) were used at 1∶10,000 dilutions to confirm equal lysate loading across samples. His-tagged HIV-1 CA was detected with 1∶15,000 α-his HRP (Clontech). CA-NC protein was detected with 1∶5,000 mouse anti-p24 antibody ab9071 (Abcam). Histone H3 was detected with 1∶2,000 rabbit histone H3 antibody #9715 (Cell Signaling Technology). All mouse and rabbit primary antibodies were detected using 1∶10,000 dilutions of anti-mouse or anti-rabbit HRP secondary antibodies (Dako).

### Immunofluorescence confocal microscopy

Cells transduced with empty LPCX vector or stably expressing HA-epitope tagged rhTrim5α, NUP153_C_, or fusion proteins thereof, were cultured on eight-well chamber slides. After 24 h, the cells were fixed with 4% paraformaldehyde for 10 min, washed, and permeabilized with PBS containing 0.5% Triton X-100. The permeabilized cells were blocked with PBS containing 10% FBS for 1 h, and stained with a 1∶100 dilution of anti-HA antibody 16b12. After a 30 min wash with PBS, the cells were incubated for 1 h with a 1∶1,000 dilution of an Alexa Fluor 555 conjugated goat anti-mouse IgG antibody (Invitrogen), as well as Hoechst 33342 (Invitrogen) diluted to a concentration of 0.2 µg/ml. After an additional 30 min wash with PBS, the samples were covered with mounting medium [150 mM NaCl, 25 mM Tris pH 8.0, 0.5% N-propyl gallate, and 90% glycerol]. The processed samples were analyzed on a Nikon Eclipse spinning disk confocal microscope.

### NUP153 protein purification

GST-NUP153_C_ was expressed in BL21-CodonPlus (DE3)-RILP *E. coli* (Agilent) grown in 2× YT media and induced at an optical density of 0.8 at 600 nm (OD_600_) with 1 mM isopropyl β-D-1-thiogalactopyranoside (IPTG) for 1 h at 18°C. Cells were pelleted at 6,000× g, and sonicated for 5 min in buffer A. The lysate was centrifuged for 30 min at 35,000× g, and the pellet was resuspended in buffer B [1 M NaCl, 25 mM Tris-HCl pH 7.5, 1 mM DTT, 1 mM EDTA, Complete protease inhibitor] with a dounce homogenizer. The lysate was again spun at 35,000× g, and the pellet was resuspended in Buffer C [2 M Urea, 200 mM NaCl, 25 mM Tris-HCl pH 7.5, 1 mM DTT, 1 mM EDTA, Complete protease inhibitor] with a dounce homogenizer. After a last centrifugation at 35,000× g, the supernatant was collected and incubated with glutathione-sepharose beads (GE Healthcare) overnight at 4°C. The beads were washed with buffer D [200 mM NaCl, 25 mM Tris-HCl pH 8.0, 1 mM DTT, 1 mM EDTA, Complete protease inhibitor], and the protein was eluted with buffer D containing 20 mM glutathione. Eluted protein was dialyzed against buffer D to remove excess glutathione, spin concentrated by ultrafiltration through a 10,000 nominal molecular weight limit (NMWL) Amicon filter (Millipore), and flash frozen in liquid nitrogen for storage at −80°C.

BL21-CodonPlus (DE3)-RILP *E. coli* transformed with pGEX2T-his-GST-pp-NUP153_C_ was grown to an OD_600_ of 0.8, followed by induction with 1 mM IPTG for 1 h at 18°C. Cells were pelleted at 6,000× g, and sonicated for 5 min in buffer A. The lysate was then centrifuged for 30 min at 35,000× g, and the pellet was resuspended in buffer E [6 M Urea, 200 mM NaCl, 25 mM Tris-HCl pH 7.5, 1 mM DTT, 1 mM EDTA, Complete protease inhibitor] with a dounce homogenizer. The lysate was then centrifuged at 40,000× g for 1 h, and the resulting supernatant was incubated with Ni-NTA conjugated agarose beads (Qiagen) overnight. The beads were then initially washed with buffer E, and then progressive dilutions of buffer E into cleavage buffer [150 mM NaCl, 50 mM Tris-HCl pH 7, 1 mM DTT, 1 mM EDTA] (3∶1, 1∶1, 1∶3), with a final wash in cleavage buffer only, each supplemented with 7.5 mM imidazole. The beads were incubated with 5 U of PreScission protease (GE Healthcare) for 48 h. The supernatant, which was cleared with 0.1 volumes of Ni-NTA beads and glutathione-sepharose beads each at 4°C to remove uncleaved protein and residual PreScission protease, was centrifuged at 21,000× g for 15 min at 4°C. The resulting supernatants were quantitated following fractionation by sodium dodecyl sulfate-polyacrylamide gel electrophoresis (SDS-PAGE) and staining with SYPRO Ruby (Invitrogen) or Coomassie blue, as compared to a standard curve of bovine serum albumin (BSA), using ChemiDoc MP imager (Bio-Rad) with Image Lab software. Cleaved full length NUP153_C_ was recovered at ∼50% purity, with the predominant contaminants degradation products of the full-length protein, as inferred through comparison with western blots using mab414 antibody.

### CA binding assays

Recombinant HIV-1 CA-NC was expressed in *E. coli*, purified, and assembled into CA-NC complexes as previously described [29]. Expression constructs encoding full-length HA-NUP153 or fragments thereof were transiently transfected into 293T cells using X-tremeGENE 9 DNA transfection reagent (Roche). Cells were collected after 48 h, lysed with successive freeze thaws in buffer H, and clarified by centrifugation at 21,000× g at 4°C. CA-NC complexes were incubated with clarified lysates for 1 h at room temperature before ultracentrifugation for 30 min at 100,000× g through a 50% sucrose cushion prepared in PBS. The resulting pellet was resuspended in 1× sample loading buffer, and fractionated by SDS-PAGE. Experiments with purified proteins were stained with Coomassie blue or SYPRO Ruby, while experiments using a lysate component were developed by western blot. Quantification was performed with a ChemiDoc MP imager using Image Lab software.

His-tagged HIV-1, MLV, EIAV, and FIV capsid proteins, either full length or NTD only, were expressed in BL21-CodonPlus (DE3)-RILP *E. coli*, grown to an OD_600_ of 0.6, and induced for 4 h with 1 mM IPTG. Bacteria pelleted by centrifugation were resuspended in Buffer A, sonicated, and centrifuged at 30,000× g for 30 min. The supernatants were incubated overnight with Ni-NTA-sepharose beads, eluted with 20 mM Tris-HCl pH 8.0, 200 mM imidazole elution buffer, and dialyzed into Tris Buffer (20 mM Tris-HCl pH 8.0). Dialyzed protein was concentrated by ultrafiltration through a 10,000 NMWL filter, centrifuged at 21,000× g, and the resulting soluble protein was quantitated by spectrophotometer.

Pull-down assays with full-length CA or CA_N_ proteins were performed by mixing 20 µl reactions with the following final concentrations: 0.02 µl packed volume Ni-NTA beads per µl (0.4 µl total), 20 µM CA, 25 mM Tris-HCl pH 8.0, and either 0.5 µM purified NUP153_C_ with 0.1% NP-40 and 150 mM NaCl, or 100 µg 293T lysate overexpressing HA-tagged NUP153 with 0.25% Triton X-100 and 200 mM NaCl. Mixtures were left rocking at room temperature for 1 h after which the samples were washed twice in buffer M [25 mM Tris-HCl pH 8.0, 150 mM NaCl, and 0.1% NP-40], allowing the beads to settle by gravity, and finally resuspended in 1× sample loading buffer. Saturation curves were achieved by mixing 3 µl packed volume Ni-NTA beads with 0.5 µM purified WT or mutant NUP153_C_, 150 mM NaCl, 25 mM Tris-HCl pH 8.0, and 0.1% NP-40, with half-log increments of HIV-1 CA_N_ from 2 µM to 200 µM. Both bead-bound and supernatant fractions were separated by SDS-PAGE and stained with SYPRO Ruby, with the percent of NUP153_C_ protein bound calculated at each concentration. The K_d_ of NUP153_C_ binding was calculated by subtracting nonspecific binding to beads and fitting the resulting data-points with a one-site saturation binding nonlinear regression using Prism6 software (GraphPad).

CPSF6 competition experiments were performed through modification of the CA-NC protocol. Assembled CA-NC was diluted to a final concentration of 0.8 µM in the reaction mixture. WT or mutant NUP153_C_ was added to a final concentration of 4 µM, along with 10 µg total 293T extract expressing C-terminally HA-tagged CPSF6, resulting in final concentrations of 170 mM NaCl, 75 mM Tris-HCl pH 8.0, and 0.025% Triton X-100. Mixtures (20 µl) were incubated at room temperature for 20 min, after which they were spun over a 30 µl 20% sucrose cushion in a microcentrifuge at 21,000× g for 20 min at 4°C. The resulting pellet was resuspended in sample loading buffer and separated by SDS-PAGE. Western blotting with p24 antibody indicated ∼35% of input CA-NC was recovered in the pellet. CA-NC co-sedimentation assays with WT or FG mutant NUP153_C_ were performed similarly, but were instead centrifuged over a 25% sucrose cushion.

### IN pull-down assay

His-tagged HIV-1 and FIV IN [84] and GST [90] were expressed and purified as previously described. Pull-down of soluble IN was performed as previously described for GST-LEDGF_326–530_ [59], with 0.8 µM of his-tagged HIV-1 or FIV IN incubated with 0.47 µM GST-NUP153_C_ or control GST pre-bound to glutathione-sepharose beads in PD buffer [150 mM NaCl, 25 mM Tris-HCl pH 7.4, 5 mM MgCl_2_, 5 mM DTT, 0.1% NP-40]. BSA (5 µg) was included as an additional specificity control. The reaction was incubated for 2 h at 4°C, after which the beads were washed 4 times with PD buffer, and settled each time for 20 min in the absence of centrifugation. Recovered samples were resolved by SDS-PAGE, and stained with Coomassie blue and western blotted with anti-his antibody.

### Statistical analysis

Dependencies between variables were assessed by Spearman rank correlation using Prism6 software. The significances of pair-wise differences were calculated by Student's t-test (two-tailed) using Prism6 software.

## Supporting Information

Figure S1 **Trim-NUP153_C_ localizes to the cell cytoplasm and restricts HIV-1 reverse transcription.** (**A**) Immunofluorescence confocal microscopy of HOS cells transduced with empty vector or the indicated HA-tagged construct. Hoescht 33342 stains DNA and therefore highlights cell nuclei. (**B**) Fractionation of rhTrim5α-HA, Trim-HA-NUP153_C_, and HA-NUP153_C_ expressing cells. Gels were probed with antibodies against the HA tag (top panels), histone H3, α-tubulin, or NUP62 (bottom panels). Cytoplasmic α-tubulin and nucleus-associated NUP62 and histone H3 marker proteins were predominantly found in fractions 1 and 3, respectively. Asterisks mark bands that correspond to the expected mobilities of full-length constructs. (**C–E**) Levels of R-U5 DNA synthesis (early reverse transcripts) (C), U5-*gag* DNA synthesis (late reverse transcripts) (D), and 2-LTR circle formation (E) in cells transduced with empty vector, rhTrim5α-HA, or Trim-HA-NUP153_C_ expression constructs at 1, 6, 24, and 48 h post HIV-1 infection, as detected by quantitative PCR. Results (averages of three experiments, with error bars denoting standard error) were normalized to levels of peak DNA amplification, which was set at 100%. (**F**) Corresponding infectivity of GFP reporter viruses, measured 48 h post infection. Data were normalized to infectivity in cells transduced with empty expression vector. Results are an average of three experiments, with error bars denoting standard error.
(TIF)

Figure S2 **Pull-down of HA-NUP153_C_ by HIV-1 CA_N_ proteins.** HA-NUP153_C_ in 293T cell lysates pulled-down by WT or various mutant his-tagged HIV-1 CA_N_ proteins, with recovered protein resolved by SDS-PAGE and detected by 3F10 and anti-his antibodies. Results are an average of 4 experiments, with error bars denoting standard error. A representative western blot result is shown. The dotted line highlights the level of HA-NUP153_C_ binding to WT CA_N_ protein.
(TIF)

Figure S3 **HIV-1 CA mutant sensitivity to CPSF6_358_ expression.** Percent infectivity of CA mutant viruses on CPSF6_358_ expressing HOS cells compared to mock transduced cells. Results are the average of 3 experiments, with error bars denoting standard error.
(TIF)

Figure S4 **GST-NUP153_C_ pull-down of HIV-1 and FIV IN.** GST-NUP153_C_ pulled-down an average of ∼0.85% of input his-tagged HIV-1 IN and ∼5.55% of input his-tagged FIV IN over 3 experiments. A representative experiment is shown. Note preferential western blot detection of the FIV IN N-terminal his-tag over that of the HIV-1 tag.
(TIF)

Figure S5 **Location of NUP153_C_ binding site within multimerized CA.** (**A**) Model of the HIV-1 CA hexamer (pdb: 3j34) [32], with surface representations of two adjacent CA units shown. Side chains involved in NUP153_C_ binding are shown as sticks and labeled, with the binding pocket highlighted by a dashed white circle. (**B**) Model of the HIV-1 inter-hexameric CA interface (pdb: 3j34). The two molecules in panel A were rotated 90° around the y-axis, −10° around the x-axis, and juxtaposed with two CA molecules from the adjacent hexamer. The z-plane was clipped to expose the NUP153_C_ binding site within the interface.
(TIF)
