# Supplementary figures and images for: Nucleoporin NUP153 Phenylalanine-Glycine Motifs Engage a Common Binding Pocket within the HIV-1 Capsid Protein to Mediate Lentiviral Infectivity
Source: PLoS Pathog. 2013 Oct 10;9(10):e1003693. doi: 10.1371/journal.ppat.1003693 (PMC3795039; doi:10.1371/journal.ppat.1003693)

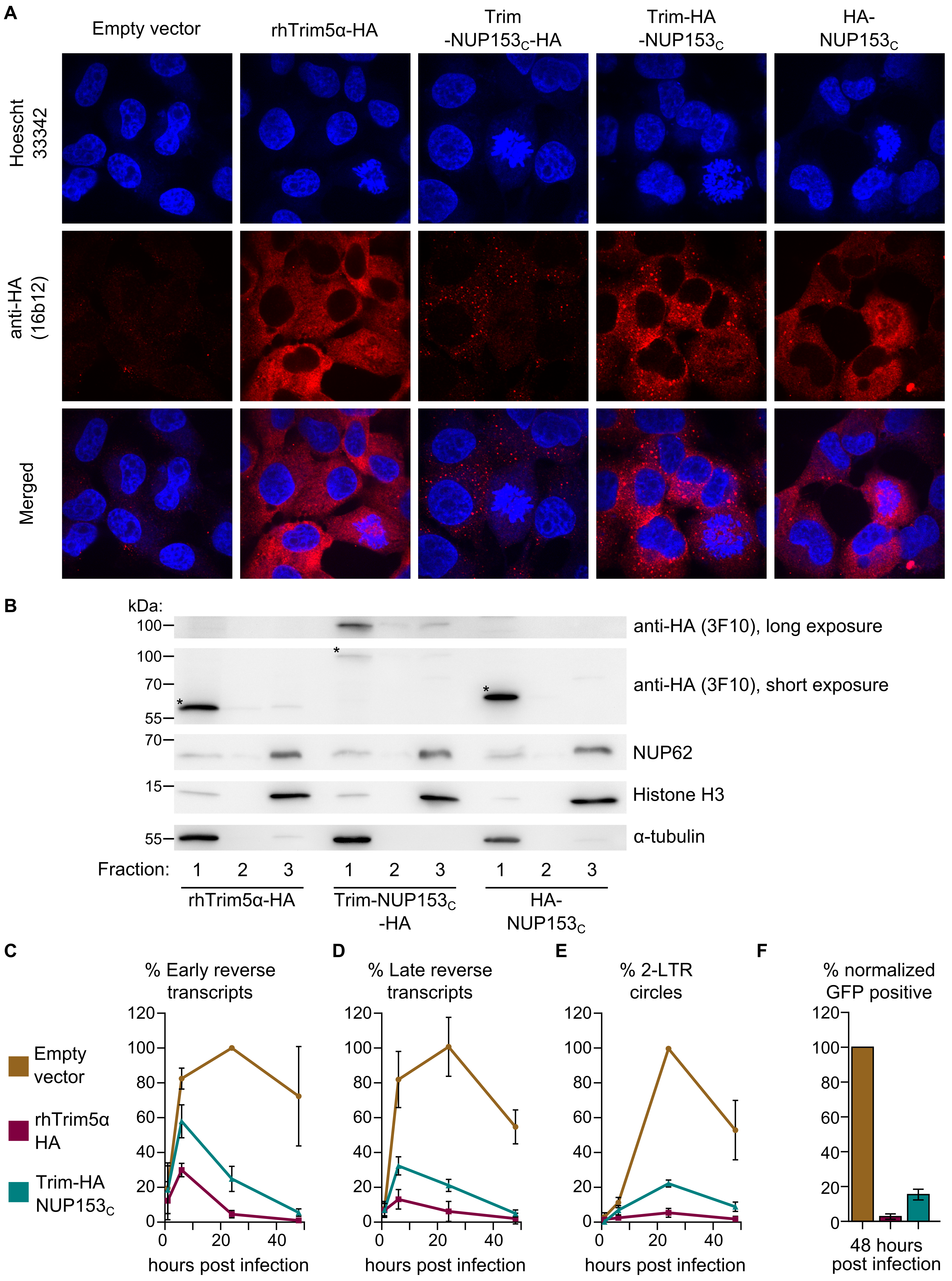

Supplement: Figure S1 — Trim-NUP153C localizes to the cell cytoplasm and restricts HIV-1 reverse transcription. (A) Immunofluorescence confocal microscopy of HOS cells transduced with empty vector or the indicated HA-tagged construct. Hoescht 33342 stains DNA and therefore highlights cell nuclei. (B) Fractionation of rhTrim5α-HA, Trim-HA-NUP153C, and HA-NUP153C expressing cells. Gels were probed with antibodies against the HA tag (top panels), histone H3, α-tubulin, or NUP62 (bottom panels). Cytoplasmic α-tubulin and nucleus-associated NUP62 and histone H3 marker proteins were predominantly found in fractions 1 and 3, respectively. Asterisks mark bands that correspond to the expected mobilities of full-length constructs. (C–E) Levels of R-U5 DNA synthesis (early reverse transcripts) (C), U5-gag DNA synthesis (late reverse transcripts) (D), and 2-LTR circle formation (E) in cells transduced with empty vector, rhTrim5α-HA, or Trim-HA-NUP153C expression constructs at 1, 6, 24, and 48 h post HIV-1 infection, as detected by quantitative PCR. Results (averages of three experiments, with error bars denoting standard error) were normalized to levels of peak DNA amplification, which was set at 100%. (F) Corresponding infectivity of GFP reporter viruses, measured 48 h post infection. Data were normalized to infectivity in cells transduced with empty expression vector. Results are an average of three experiments, with error bars denoting standard error. (TIF) [file ppat.1003693.s001.tif]

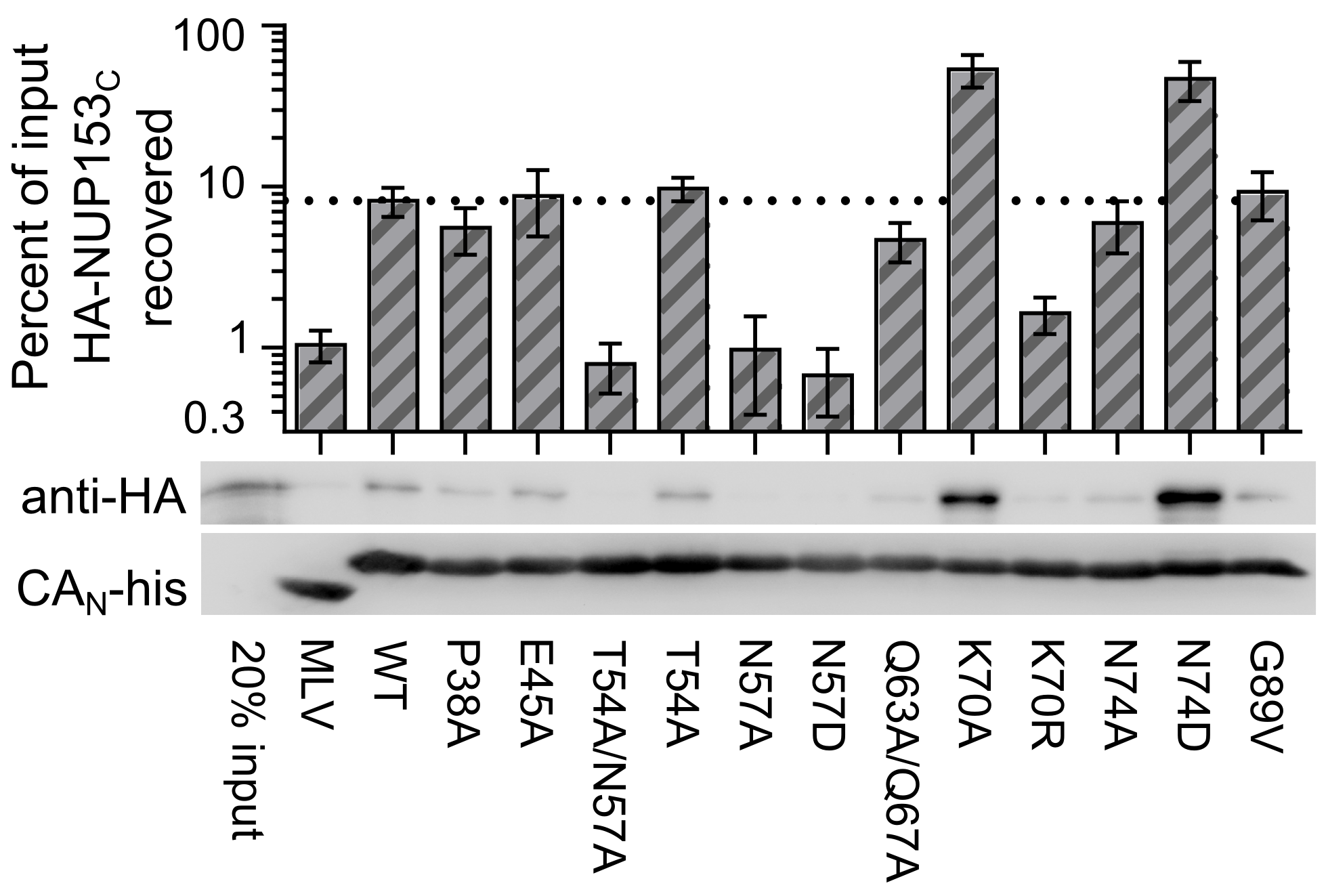

Supplement: Figure S2 — Pull-down of HA-NUP153C by HIV-1 CAN proteins. HA-NUP153C in 293T cell lysates pulled-down by WT or various mutant his-tagged HIV-1 CAN proteins, with recovered protein resolved by SDS-PAGE and detected by 3F10 and anti-his antibodies. Results are an average of 4 experiments, with error bars denoting standard error. A representative western blot result is shown. The dotted line highlights the level of HA-NUP153C binding to WT CAN protein. (TIF) [file ppat.1003693.s002.tif]

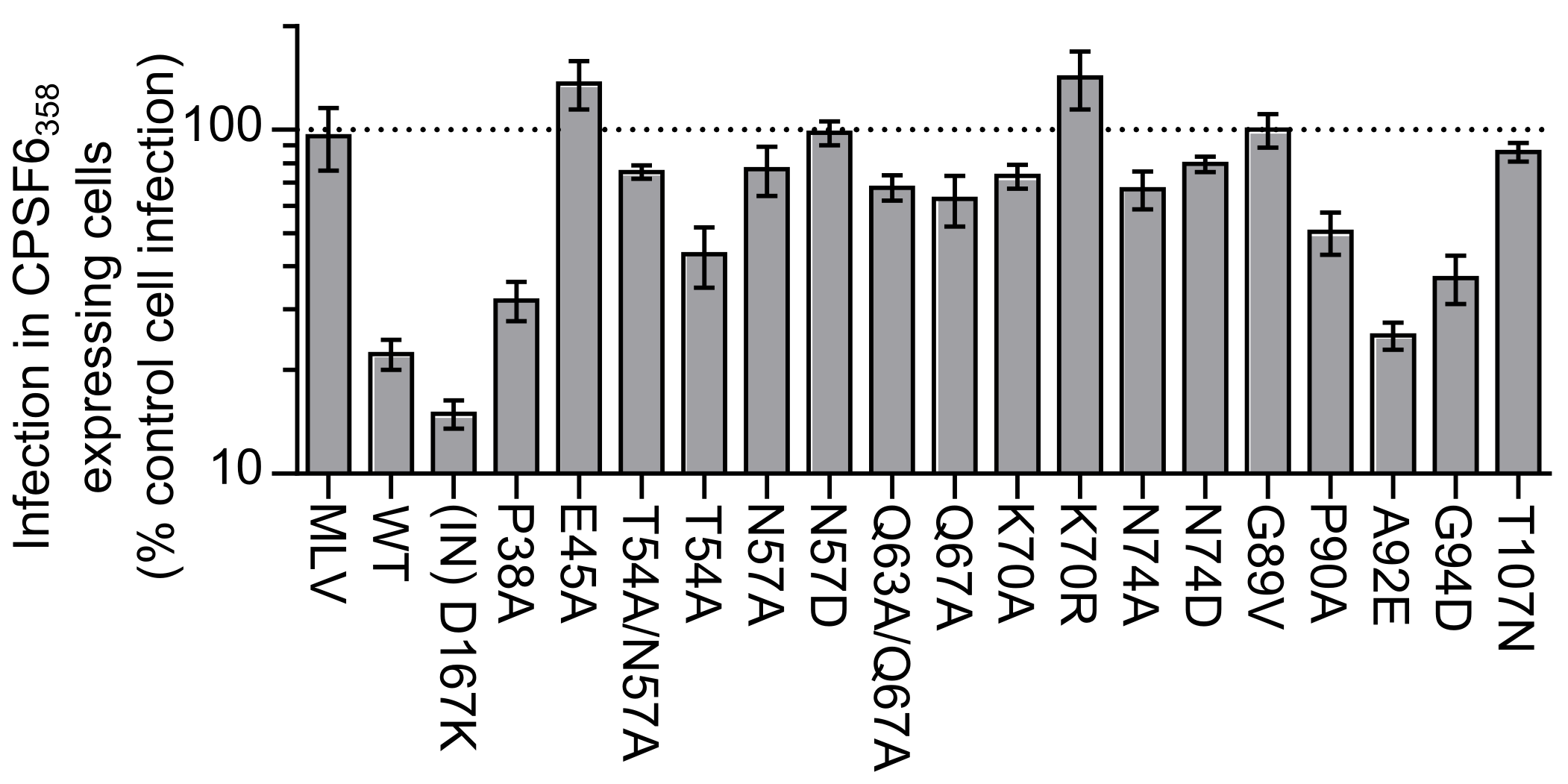

Supplement: Figure S3 — HIV-1 CA mutant sensitivity to CPSF6358 expression. Percent infectivity of CA mutant viruses on CPSF6358 expressing HOS cells compared to mock transduced cells. Results are the average of 3 experiments, with error bars denoting standard error. (TIF) [file ppat.1003693.s003.tif]

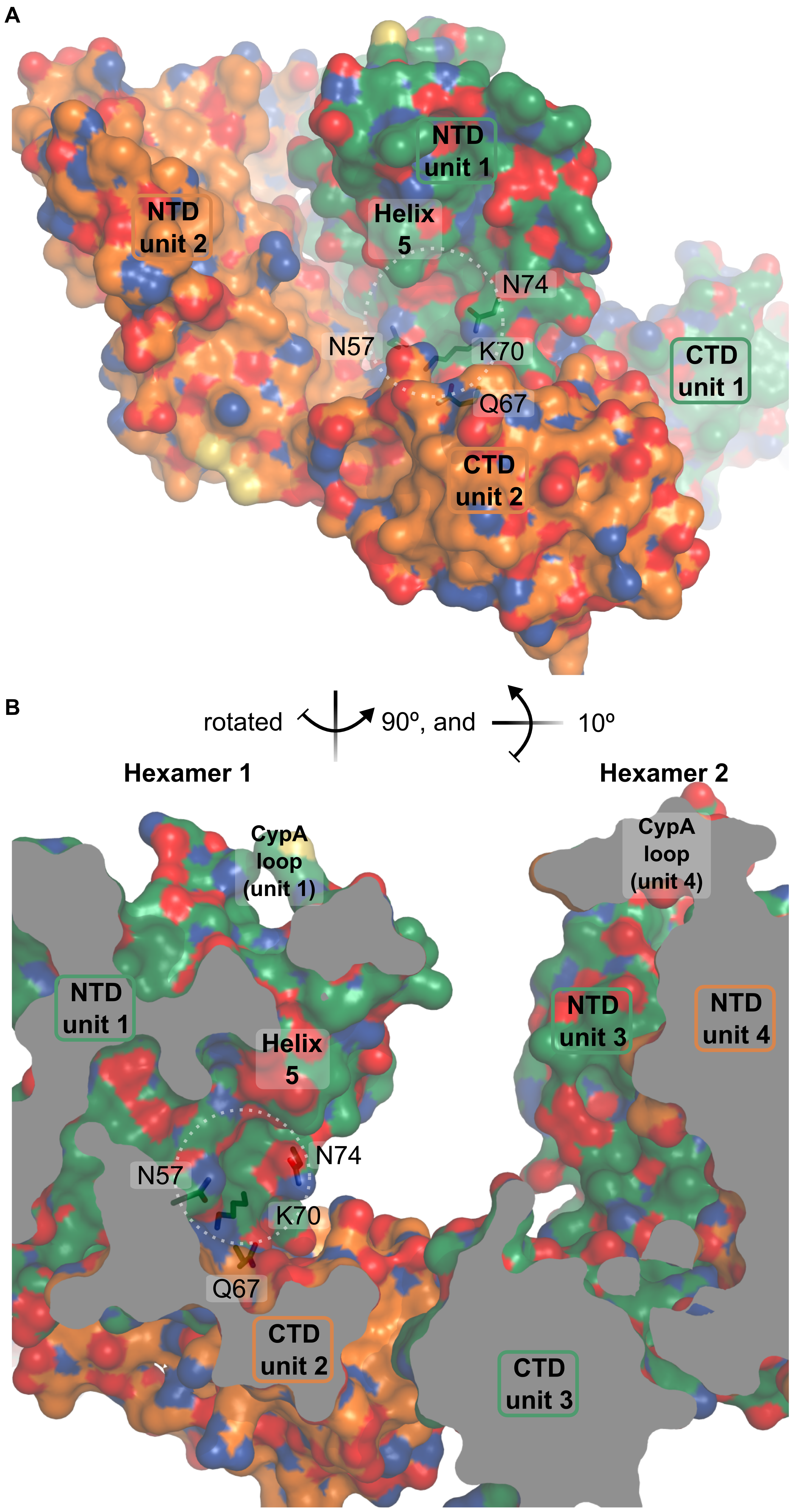

Supplement: Figure S5 — Location of NUP153C binding site within multimerized CA. (A) Model of the HIV-1 CA hexamer (pdb: 3j34) [32], with surface representations of two adjacent CA units shown. Side chains involved in NUP153C binding are shown as sticks and labeled, with the binding pocket highlighted by a dashed white circle. (B) Model of the HIV-1 inter-hexameric CA interface (pdb: 3j34). The two molecules in panel A were rotated 90° around the y-axis, −10° around the x-axis, and juxtaposed with two CA molecules from the adjacent hexamer. The z-plane was clipped to expose the NUP153C binding site within the interface. (TIF) [file ppat.1003693.s005.tif]
